# The proteogenomic landscape of multiple myeloma reveals insights into disease biology and therapeutic opportunities

**DOI:** 10.1038/s43018-024-00784-3

**Published:** 2024-06-28

**Authors:** Evelyn Ramberger, Valeriia Sapozhnikova, Yuen Lam Dora Ng, Anna Dolnik, Matthias Ziehm, Oliver Popp, Eric Sträng, Miriam Kull, Florian Grünschläger, Josefine Krüger, Manuela Benary, Sina Müller, Xiang Gao, Arunima Murgai, Mohamed Haji, Annika Schmidt, Raphael Lutz, Axel Nogai, Jan Braune, Dominik Laue, Christian Langer, Cyrus Khandanpour, Florian Bassermann, Hartmut Döhner, Monika Engelhardt, Christian Straka, Michael Hundemer, Dieter Beule, Simon Haas, Ulrich Keller, Hermann Einsele, Lars Bullinger, Stefan Knop, Philipp Mertins, Jan Krönke

**Affiliations:** 1 Charité – Universitätsmedizin Berlin, Corporate member of Freie Universität Berlin and Humboldt-Universität zu Berlin, Berlin, Germany; 2 Max Delbrück Center for Molecular Medicine, Berlin, Germany; 3 German Cancer Consortium (DKTK), partner site Berlin, DKFZ and Charité – Universitätsmedizin Berlin, Berlin, Germany; 4 Berlin Institute of Health, Berlin, Germany; 5 Internal Medicine III, University Hospital Ulm, Ulm, Germany; 6 German Cancer Research Center (DKFZ), Heidelberg, Germany; 7 Heidelberg Institute for Stem Cell Technology and Experimental Medicine, Heidelberg, Germany; 8 Faculty of Biosciences, Heidelberg University, Heidelberg, Germany; 9 Department of Medicine V, Hematology, Oncology and Rheumatology, Heidelberg University Hospital, Heidelberg, Germany; 10 Department of Medicine A, Hematology, Oncology and Pneumology, University Hospital Muenster, Muenster, Germany; 11 Department of Medicine III, Technical University of Munich, Klinikum rechts der Isar, Munich, Germany; 12 Freiburg University Hospital, Freiburg, Germany; 13 Medizinische Klinik, München Klinik Schwabing, Munich, Germany; 14 Department of Internal Medicine II, University Hospital Würzburg, Würzburg, Germany; 15 Nuremberg General Hospital, Nuremberg, Germany; 16 Paracelsus Medical School, Nuremberg, Germany

**Keywords:** Cancer, Cancer genomics, Proteomics, Next-generation sequencing

## Abstract

Multiple myeloma (MM) is a plasma cell malignancy of the bone marrow. Despite therapeutic advances, MM remains incurable, and better risk stratification as well as new therapies are therefore highly needed. The proteome of MM has not been systematically assessed before and holds the potential to uncover insight into disease biology and improved prognostication in addition to genetic and transcriptomic studies. Here we provide a comprehensive multiomics analysis including deep tandem mass tag-based quantitative global (phospho)proteomics, RNA sequencing, and nanopore DNA sequencing of 138 primary patient-derived plasma cell malignancies encompassing treatment-naive MM, plasma cell leukemia and the premalignancy monoclonal gammopathy of undetermined significance, as well as healthy controls. We found that the (phospho)proteome of malignant plasma cells are highly deregulated as compared with healthy plasma cells and is both defined by chromosomal alterations as well as posttranscriptional regulation. A prognostic protein signature was identified that is associated with aggressive disease independent of established risk factors in MM. Integration with functional genetics and single-cell RNA sequencing revealed general and genetic subtype-specific deregulated proteins and pathways in plasma cell malignancies that include potential targets for (immuno)therapies. Our study demonstrates the potential of proteogenomics in cancer and provides an easily accessible resource for investigating protein regulation and new therapeutic approaches in MM.

## Abstract (web summary)

Krönke and colleagues present a multiomic resource of plasma cell malignancies, including multiple myeloma, that comprises phosphoproteomics, RNA and DNA sequencing and provides insights into cancer type biology and candidate therapeutic targets.

## Main

Multiple myeloma (MM), the second most frequent hematologic malignancy, is characterized by expansion of monoclonal plasma cells in the bone marrow. Patients suffer from bone lesions, renal insufficiency, hypercalcemia and bone marrow failure^1^. The introduction of effective therapies including thalidomide analogs, proteasome inhibitors and immunotherapies such as chimeric antigen receptor (CAR)-T cells in the past decade substantially extended survival in MM. However, MM is still considered incurable and those patients with high-risk characteristics have a particularly poor outcome^1^.

Chromosomal alterations are the initiating step in the pathogenesis of MM that are already present in the premalignant stage of monoclonal gammopathy of undetermined significance (MGUS). Primary genetic events define the cytogenetic subgroups of MM^2^ and are associated with a distinct gene expression profile^3,4^. Half of the patients exhibit translocations involving the immunoglobulin heavy chain (*IgH*) enhancer on chromosome 14, predominantly with oncogenes *CCND1* (t(11;14)), *NSD2* and *FGFR3* (t(4;14)) and *MAF B* (t(14;16)). Patients without these translocations typically have a hyperdiploid (HRD) karyotype with trisomies primarily of the odd-numbered chromosomes. Secondary genetic events occur later in the pathogenesis of MM and include del(13q) comprising *RB1*, del(17p) comprising *TP53*, gain or amplification of chromosome 1q and mutations in *NRAS*, *KRAS*, *TP53, TENT5C* (*FAM46C*) and *DIS3* (refs. ^5–7^). Genetics together with blood protein levels of albumin, b2-microglobulin and lactate dehydrogenase are incorporated in the revised international staging system (R-ISS), the current standard for risk classification and therapy stratification in MM^6^.

Proteomics has recently emerged as a technology to study cancer biology, generate prognostic and predictive models and identify new therapeutic targets^8^. Proteogenomic studies integrating genomics and transcriptomics in solid tumors^9–11^ and in hematologic malignancies^12–14^ revealed low correlation between RNA and protein expression, demonstrating that inferring the activity of proteins merely based on studying RNA expression is limited. While many proteogenomic studies contribute to the general understanding of disease mechanisms, only a few of them have connected proteome alterations to clinical outcome^10,12,15,16^. For MM, a limited number of proteomic studies have been conducted in small cohorts^17–21^, while comprehensive proteogenomic studies that evaluate how the proteome is influenced by genetic alterations, disease stage and how protein expression impact outcomes, are currently missing. In this Resource, to address this gap, we performed an integrated multiomics study, including tandem isobaric mass tag (TMT)-based quantitative global- and phosphoproteomic analysis, RNA sequencing and whole-genome nanopore DNA sequencing to assess copy number alterations (CNAs) of 138 patients with plasma cell malignancies of different disease stages including MGUS, newly diagnosed multiple myeloma (NDMM) and plasma cell leukemia (PCL), a highly aggressive form of plasma cell dyscrasias.

## Results

### Proteomic landscape of newly diagnosed MM

**Fig. 1:**
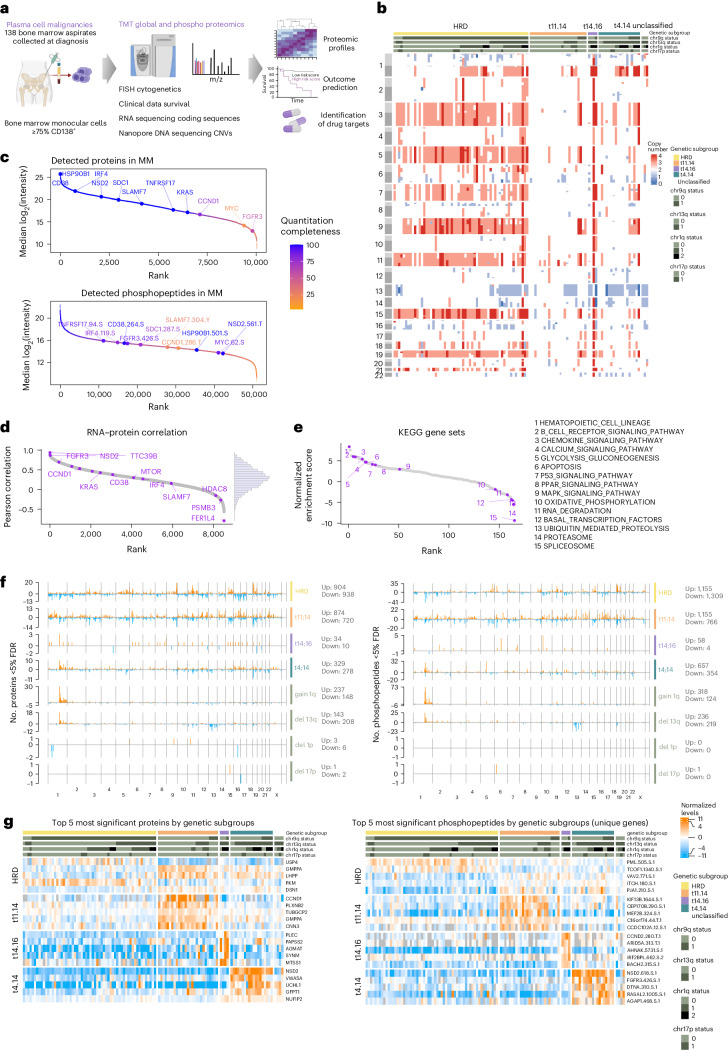
Proteogenomic landscape of newly diagnosed MM. **a**, Overview of the proteogenomic study. **b**, A heat map of CNVs detected by nanopore sequencing in 109 cases of NDMM sorted by primary genetic subgroup: HRD, t(11;14), t(4;14) and t(14;16) translocations. Cytogenetic alterations, including deletions, amplifications and translocations were detected by FISH. **c**, Proteins and phosphopeptides detected by TMT-based mass spectrometry ranked by median intensity. **d**, Ranked gene symbol-wise Pearson correlation of mRNA–protein levels across MM samples (*n* = 8,511 RNA–protein pairs with at least ten valid values in both datasets). **e**, ssGSEA of the mRNA–protein correlations for KEGG pathways (*n* = 165 ranked pathways). Gene sets were ranked by their normalized enrichment score and informative pathways are annotated with purple circles. **f**, Differentially regulated proteins (left) and phosphopeptides (right) in each cytogenetic subgroup were determined with a two-sided, moderated two-sample *t*-test comparing subsets of samples against all other samples. The number of significant hits (FDR <0.05) in each group is plotted across genomic location. **g**, Heat maps displaying the five most significant proteins (left) and phosphopeptides (right) in each genetic subgroup across MM samples. For phosphopeptides mapping to the same protein, only the most significant entry is displayed. Phosphopeptides are annotated with gene name, position, amino acid and number of phosphorylations present. Source data

**Extended Data Fig. 1:**
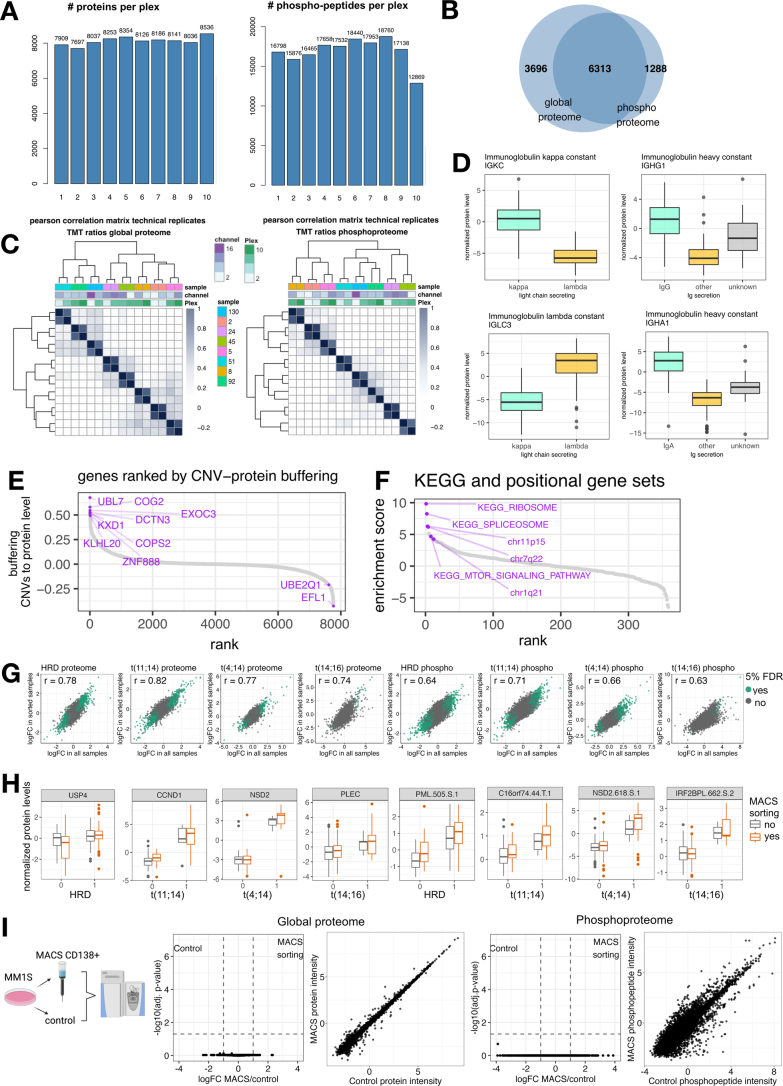
Quality control and influence of cell sorting. **a**: Numbers of proteins and phosphopeptides detected in each TMT plex. **b**: Overlap of detected protein IDs in the proteome and phosphoproteome datasets. **c**: Correlation matrix showing Pearson correlation of technical replicates (normalized TMT ratios). **d**: Immunoglobulin constant light chain protein levels. Predominant light chain kappa n = 83, lambda n = 39. Predominant immunoglobulin constant IgG n = 68, IgA n = 32; other n = 24, unknown n = 14. Boxplots show median (middle line), 25th and 75th percentiles, whiskers extend to minimum and maximum excluding outliers (values outside of 1.5 times the interquartile range (IQR)). **e**: Genes ranked by the buffering score of CNVs from RNA to protein level. The buffering score was calculated with a customized score and for each gene (g) the Pearson correlation of protein to copy number (CN) was subtracted from the Pearson correlation of RNA to CN. The resulting delta was corrected with the average copy number effect diverging from a diploid genotype. Genes are ranked from highest (high buffering of CNVs from RNA to protein level) to lowest score. **f**: SsGSEA of the protein-CNV buffering score in S1E for KEGG and positional pathways (*n* = 359 ranked pathways) showing that CNVs of certain pathways are buffered from RNA to protein level. **g**: Correlation of protein and phosphopeptides changes in each genetic subgroup in all samples (x-axis) and MACS-sorted samples (y-axis) in MM cohort. Regulated proteins (< 0.05 FDR) are indicated in green. **h**: Levels of top-regulated proteins and phosphopeptides in each genetic subgroup in MM samples with and without MACS sorting. HRD sorted n = 35, HRD unsorted n = 25, HRDneg sorted n = 41, HRDneg unsorted n = 13; t(11.14) sorted n = 24, t(11.14) unsorted n = 3, t(11.14)neg sorted n = 52, t(11.14)neg unsorted n = 35; t(14.16) sorted n = 3, t(14.16) unsorted n = 1, t(14.16)neg sorted n = 73, t(14.16)neg unsorted n = 37; t(t4.14) sorted n = 11, t(t4.14) unsorted n = 8, t(t4.14)neg sorted n = 65, t(t4.14)neg unsorted n = 30; Boxplots show median (middle line), 25th and 75th percentiles, whiskers extend to minimum and maximum excluding outliers (values outside of 1.5*IQR). **i**: MM1S cells were sorted with CD138 + MACS and the global proteome and phosphoproteome were analyzed with label-free proteomics (n = 4, biological replicates). MACS-sorted samples were compared against the control with a moderated 2-sample t-test. No significant differences between MACS-sorted and non-sorted MM.1S cells were detected (< 0.05 FDR). Plots show results of moderated 2-sample t-test and correlation of averaged normalized intensities in both groups. Source data

To characterize the proteomic landscape of treatment-naive symptomatic MM we analyzed plasma cells isolated from 114 patients with NDMM (Fig. 1a and Supplementary Table 1). The frequency of primary and secondary chromosomal alterations, as assessed by fluorescence in situ hybridization (FISH) was distributed according to the described incidence in MM^6^. RNA sequencing (Supplementary Table 2) and nanopore whole-genome DNA sequencing (Supplementary Table 3) were conducted for the majority of samples to assess gene expression and CNAs, respectively, which largely aligned with the genetic alterations detected by FISH (Fig. 1b and Supplementary Table 3). Global proteome and phosphoproteome levels were quantified with TMT. The number of identified proteins and phosphopeptides across TMT plexes was comparable (Extended Data Fig. 1a) and in total, over 10,000 proteins and 50,000 phosphopeptides were identified, of which 8,336 proteins and 25,131 phosphopeptides were quantified in at least half of the samples (Fig. 1c). The phosphoproteomic data extended the number of detected proteins to 11,297 proteins (Fig. 1c and Extended Data Fig. 1b). Technical replicates showed a good correlation, and no batch effects of TMT plexes were observed (Extended Data Fig. 1c). Key plasma cell markers, including the transcription factor IRF4, surface proteins CD38, TNFRSF17 (BCMA) and SDC1 (CD138) and translocation partners NSD2, FGFR3 and CCND1 were identified (Fig. 1c). Immunoglobulin heavy and light chain protein levels corresponded to clinical metadata (Extended Data Fig. 1d). Compensation effects of CNAs from RNA to protein levels were especially observed for ribosomal, spliceosome and proteasome proteins as well as proteins located on 1q (Extended Data Fig. 1e,f). RNA-to-protein correlation was moderate, with a median Pearson correlation coefficient of 0.29 (Fig. 1d) and proteins affected by translocations, as well as key cell surface proteins and transcription factors, displayed above-average correlation (Fig. 1d). Single sample gene set enrichment analysis (ssGSEA) of ranked RNA–protein correlations revealed enrichment of individual signaling pathways and negative enrichment of genes associated with splicing, proteasomal degradation and oxidative phosphorylation (Fig. 1e). These data imply extensive posttranscriptional regulation in MM. We observed varying levels of immune cell signatures as contaminants arising from differences in CD138^+^ sorting status and efficiency, but these did not compromise the major distinctions we identified between the different genetic subgroups (Extended Data Fig. 1g,h). The CD138^+^ cell enrichment procedure itself had no effect on the (phospho)proteome of malignant plasma cells as assessed in the myeloma cell line MM.1S (Extended Data Fig. 1i and Supplementary Tables 4 and 5).

**Extended Data Fig. 2:**
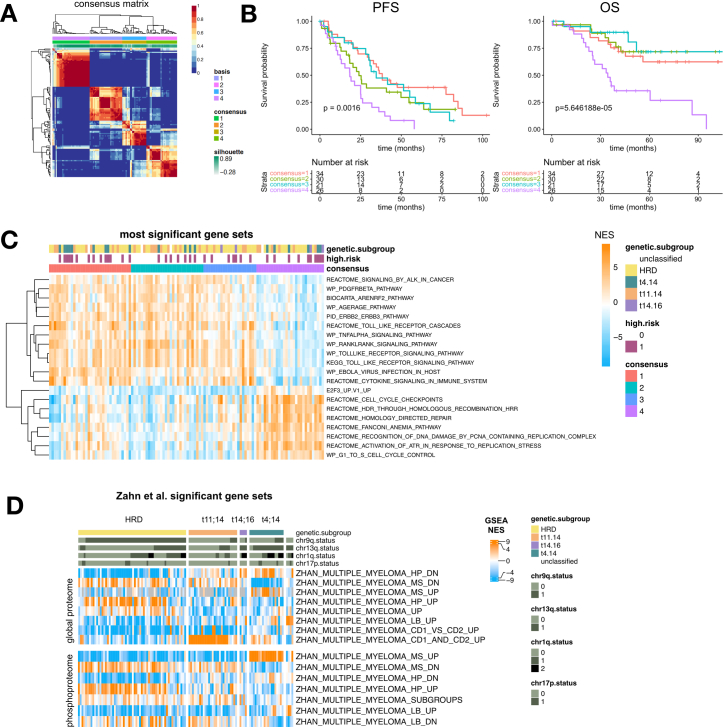
Unsupervised clustering of phosphoproteomic data. **a**: SsGSEA normalized enrichment scores of phosphoproteomic data were used as input for non-negative matrix factorization (NMF) clustering. NMF consensus map is shown. **b**: Kaplan-Meier plots show progression-free survival (PFS) and overall survival (OS) of MM patients grouped by consensus cluster as shown in A. Survival in different groups was compared with a log-rank test. **c**: Gene sets of phosphoproteomic data most significantly different between consensus cluster 4 and other clusters (moderated t-test, the 20 most significant gene sets (FDR < 0.05) are shown). **d**: TMT ratios were analyzed with ssGSEA using the gene sets C2.all.v7.0.symbols.gmt, c6.all.v7.0.symbols.gmt and h.all.v7.0.symbols.gmt. Heatmaps display ssGSEA normalized enrichment scores (NES) of Zhan et al. gene sets significant between myeloma genetic subgroups (ANOVA; FDR < 0.14). Global proteome data (top) and phosphoproteome data (bottom) are shown. Source data

Unsupervised nonnegative matrix factorization (NMF) clustering of phosphoproteomics-derived pathways (Extended Data Fig. 2a) identified a distinct subcluster of patients with lower survival probability (Extended Data Fig. 2b). This cluster was independent of genetic alterations and characterized by upregulation of proliferation and cell cycle signatures, alongside downregulation of TNF-α and ERBB signaling pathways (Extended Data Fig. 2c).

### Genetic alterations affect protein levels in *cis* and *trans*

Given the central role of chromosomal aberrations in disease initiation, biology and prognosis in MM, we determined the impact of common genetic alterations on the (phospho)proteome with differential expression analysis. Most translocations, HRD, +1q and del(13q), had a profound effect on the expression levels of proteins in *cis* and in *trans*. Less regulation was observed by t(14;16), del(1p) or del(17q) although this could in part be explained by the smaller sample numbers and thus reduced statistical power (Fig. 1f and Supplementary Tables 6 and 7). The most significant proteins and phosphopeptides in the genetic subtypes are IgH translocation partners and proteins encoded on chromosomes affected by CNAs (Fig. 1g). SsGSEA of global and phosphoproteomic data confirmed significant regulation of myeloma molecular subgroups previously defined by RNA expression studies^3^ (Extended Data Fig. 2d).

**Fig. 2:**
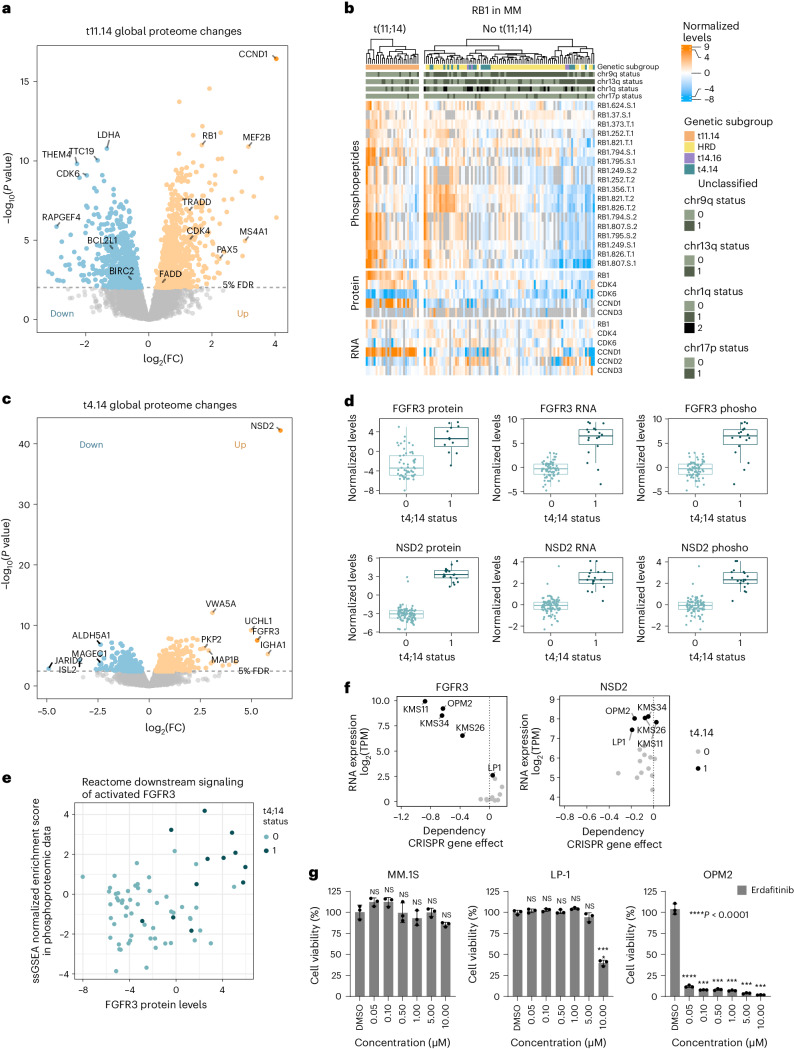
(Phospho)proteomic profiles of primary translocations t(11;14) and t(4;14). **a**, Global protein levels in newly diagnosed MM cases with t(11;14) (*n* = 27) were compared against cases without t(11;14) (*n* = 87) with a two-sided, moderated two-sample *t*-test. The log_2_ fold change (FC) of each protein is plotted against its *P* value. *P* values were adjusted with the Benjamini–Hochberg method and the significance threshold of 0.05 FDR is indicated. **b**, The heat map displays the normalized expression of RB1, CDK4, CDK6, CCND1, CCND2 and CCND3 on RNA and protein level and RB1 phosphopeptides. Phosphopeptides are annotated with protein name, position, amino acid and number of phosphorylations. **c**, Global protein levels in cases with t(4;14) (*n* = 19) were compared against other MM cases (*n* = 95) with a two-sided, moderated two-sample *t*-test. The log_2_FC of each protein is plotted against its *P* value. *P* values were adjusted with the Benjamini–Hochberg method and the significance threshold of 0.05 FDR is indicated. **d**, Protein, phosphoprotein and RNA expression levels of FGFR3 and NSD2 in samples with (*n* = 19) or without t(4;14) (*n* = 95). For phosphopeptide data, the peptide with the least missing values was selected for a graphical representation (FGFR3.S.425; NSD2.S.618). FDRs of the comparison between the two groups are indicated. Box plots show median (middle line), 25th and 75th percentiles, whiskers extend to minimum and maximum excluding outliers (values greater than 1.5× interquartile range (IQR)). **e**, FGFR3 protein levels in MM samples are plotted against the ssGSEA normalized enrichment score of the Reactome gene set ‘Downstream signaling of activated FGFR3 in phosphoproteomic data’. Normalized TMT ratios in each sample were used as input for ssGSEA. **f**, FGFR3 and NSD2 RNA expression and CRISPR–Cas9 KO screening data in MM cell lines were extracted from the depmap portal (depmap.org). RNA expression is plotted against the CRISPR KO gene effect. **g**, Cell viability of MM cell lines after treatment with FGFR3 inhibitor erdafitinib for 96 h at indicated concentrations (*n* = 3, independent replicates). Data are plotted as mean ± s.d. Drug treatments of each cell line were compared to respective DMSO controls with a Dunnett’s test. ****P value < 0.0001. Exact *P* values listed in the source table. Source data

**Extended Data Fig. 3:**
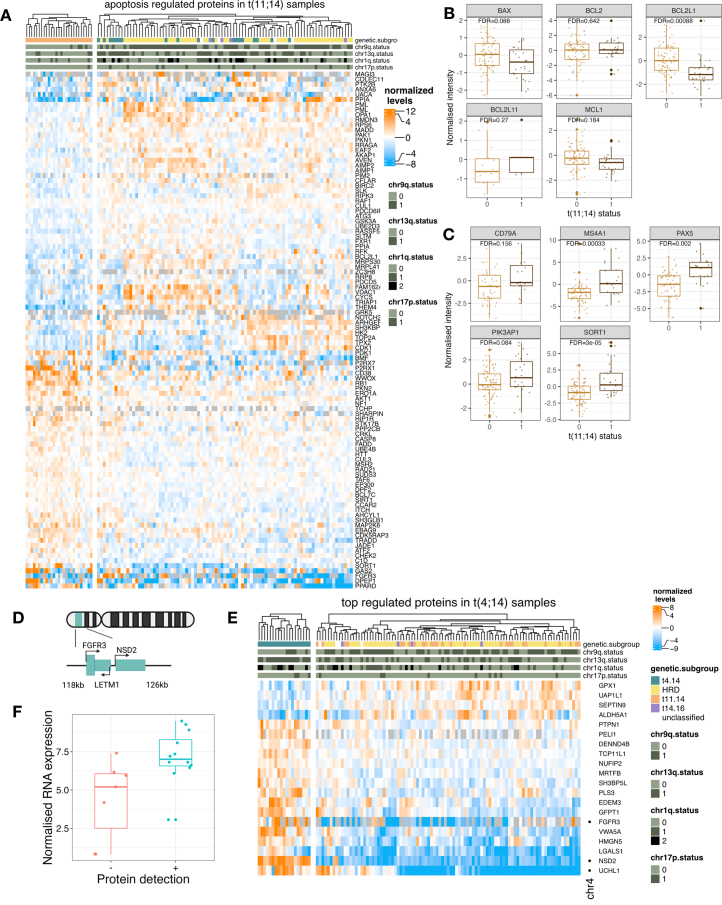
Protein level changes in multiple myeloma patients with translocations t(11;14) and t(4;14). **a**: Significantly regulated proteins in t(11;14) (FDR < 0.05) with the GO term annotation apoptosis. **b**: Levels of proteins involved in venetoclax response in patients with (n = 27) and without (n = 87) t(11;14). FDR is indicated. Boxplot shows median (middle line), 25th and 75th percentiles, whiskers extend to minimum and maximum excluding outliers (values greater than 1.5 times the interquartile range). **c**: Protein levels of selected B cell markers and genes in CD2 gene set in patients with (n = 27) and without (n = 87) t(11;14). FDR is indicated. Boxplot shows median (middle line), 25th and 75th percentiles, whiskers extend to minimum and maximum excluding outliers (values greater than 1.5 times the interquartile range). **d**: Schematic representation of the chromosomal locus on 4p16 affected by t(4;14). **e**: Levels of the most regulated proteins in t(4;14) samples (top 20 by FDR). Row annotation: dots indicate proteins located on chromosome 4. **f**: Normalized FGFR3 RNA levels in t(4;14) patients with (n = 13) or without (n = 7) FGFR3 protein detection. FDR is indicated. Boxplot shows median (middle line), 25th and 75th percentiles, whiskers extend to minimum and maximum excluding outliers (values greater than 1.5 times the interquartile range). Source data

In cases with t(11;14) cell cycle regulators were highly deregulated, including high expression of the translocation partner CCND1, increased CDK4 protein levels and RB1 phosphorylation, with concomitantly decreased CDK6 protein levels (Fig. 2a,b). In non-t(11;14) cases, high RB1 phosphorylation was instead associated with CDK6 protein expression and/or high levels of CCND2 and CCND3 RNA and phosphoprotein (Fig. 2b). T(11;14) myeloma is the only genetic subgroup sensitive to venetoclax, a selective inhibitor of BCL2 (ref. ^22^). Of note, we found 102 apoptosis-related proteins deregulated in t(11;14) myeloma, including downregulation of apoptosis inhibitor BIRC2 and BCL2L1 (BCL-XL) and upregulation of proapoptotic proteins such as TRADD and FADD (Fig. 2a and Extended Data Fig. 3a,b). We also detected elevated protein levels of several B cell markers and genes present in the myeloma CD2 gene set^3^ (Extended Data Fig. 3c), which may also be linked to BCL2 dependency in t(11;14) myeloma^3,23,24^.

**Extended Data Fig. 4:**
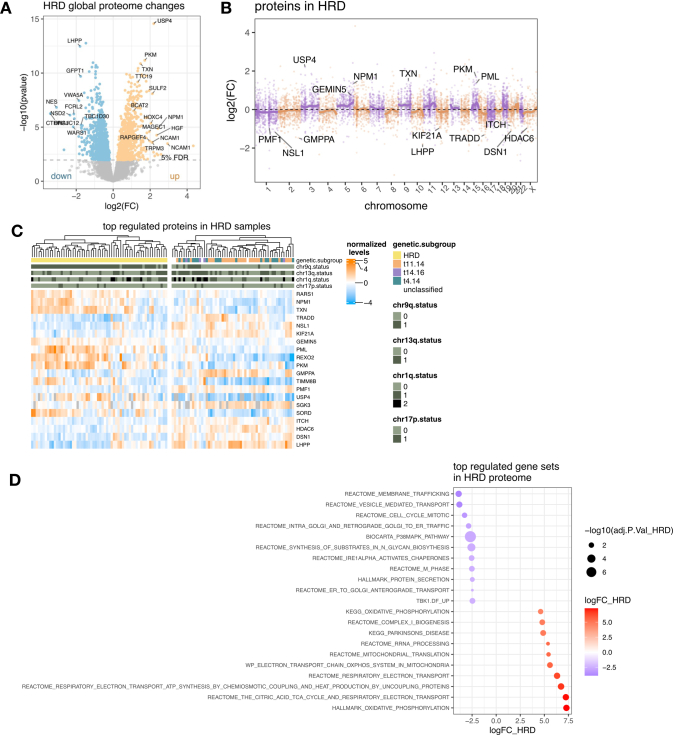
Proteins deregulated in hyperdiploid myeloma. **a**: Global protein levels in HRD samples without translocation were compared against all other samples with a 2-sided, moderated 2-sample t-test. The log2 of fold change of each protein is plotted against its p-value. P-values were adjusted with the Benjamini-Hochberg method and the significance threshold of 0.05 FDR is indicated. **b**: Log2 fold changes of proteins in HRD samples mapped to the chromosomal location. Line indicates smoothed conditional mean. The 15 most significantly regulated proteins in HRD samples are indicated by gene name. **c**: Protein levels (normalized TMT ratios) of the most regulated proteins in HRD samples (top 20 by FDR). **d**: Normalized TMT ratios were used as input for an ssGSEA with the gene sets C2.all.v7.0.symbols.gmt, c6.all.v7.0.symbols.gmt and h.all.v7.0.symbols.gmt. Normalized enrichment scores in HRD samples were compared against HRD samples with a 2-sided, moderated 2-sample t-test. The most 10 most up and down regulated significant gene sets (< 0.05 FDR) are displayed. Source data

Translocation *t(4;14)* leads to integration of the *IgH* enhancer upstream of *NSD2* (*MMSET*) and *FGFR3* (Extended Data Fig. 3d). NSD2 was selectively and strongly upregulated on RNA, protein and phosphoprotein levels in all t(4;14) patients (Fig. 2c,d). In contrast, FGFR3 was not uniformly expressed and could be detected only in 12/19 (63%) samples with t(4;14) (Fig. 2d and Extended Data Fig. 4e), consistent with RNA data (Extended Data Fig. 3f) and previous findings^25–27^. SsGSEA of phosphoproteomic data revealed upregulation of the FGFR3 signaling pathway in samples with elevated FGFR3 protein independent of t(4;14) status (Fig. 2e). FGFR3 protein expression highly correlated with dependency on FGFR3 while NSD2 knockout (KO) shows no effect on survival in MM cell lines (Fig. 2f)^28,29^. Accordingly, the FGFR inhibitor erdafitinib was highly effective in the t(4;14) positive/FGFR3 high cell line OPM2, but ineffective in FGFR3-negative cells irrespective of t(4;14) status (Fig. 2g). Among the top upregulated proteins in t(4;14) cases in *trans* is the deubiquitinating enzyme ubiquitin C terminal hydrolase L1 (UCHL1) (Fig. 2c and Extended Data Fig. 3e). UCHL1 has been previously shown to be essential for MM and other B cell malignancies and is associated with aggressive disease^30^.

In HRD myeloma cases, we detected changes in the proteome that reflect characteristic patterns of aneuploidy (Extended Data Fig. 4a,b). Most significantly upregulated proteins include the deubiquitinase USP4 (chr3), as well the redox regulator TXN (chr9) and pyruvate kinase PKM (chr15) (Extended Data Fig. 4c). Pathway analysis revealed upregulation of the tricarboxylic acid cycle cycle and oxidative phosphorylation, and downregulation of mitotic cell cycle gene signatures (Extended Data Fig. 4d).

**Extended Data Fig. 5:**
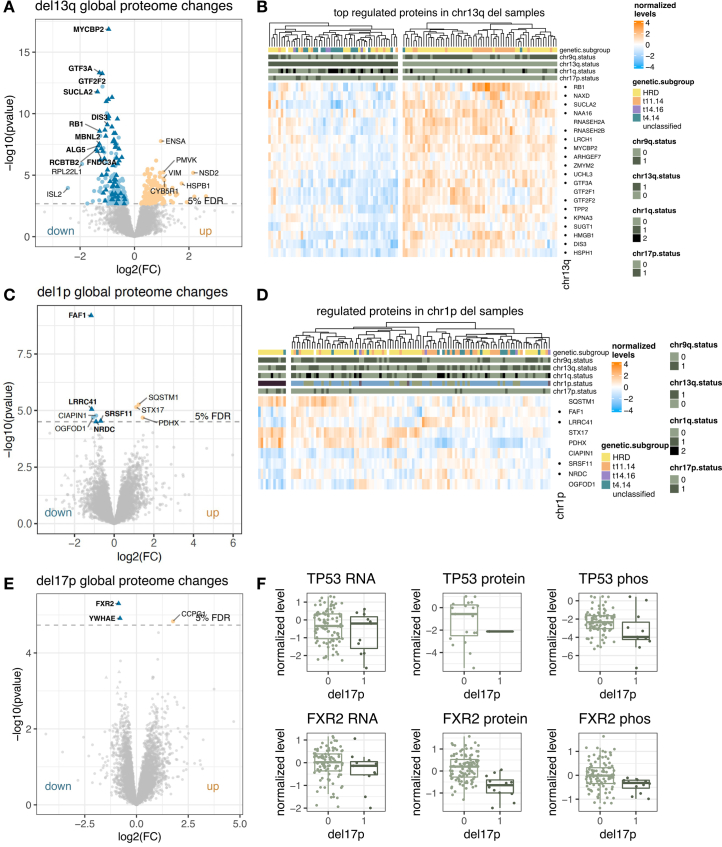
Proteins deregulated in del13q, del1p and del17p myeloma. **a**: Global protein levels in del13q samples were compared against all other samples with a 2-sided, moderated 2-sample t-test. The log2 of fold change of each protein is plotted against its p-value. P-values were adjusted with the Benjamini-Hochberg method and the significance threshold of 0.05 FDR is indicated. Proteins located on 13q are indicated with a triangle. **b**: Protein levels of the most regulated proteins in del13q samples (top 20 by FDR). Row annotation indicates proteins located on chromosome 13q. **c**: Global protein levels in del1p samples were compared against all other samples with a 2-sided, moderated 2-sample t-test. The log2 of fold change of each protein is plotted against its p-value. P-values were adjusted with the Benjamini-Hochberg method and the significance threshold of 0.05 FDR is indicated. Proteins located on 1p are indicated with a triangle. **d**: Protein levels of significantly regulated proteins in del1p samples. Row annotation indicates proteins located on chromosome 1p. **e**: Global protein levels in del17p samples were compared against all other samples with a 2-sided, moderated 2-sample t-test. The Log2 of fold change of each protein is plotted against its p-value. P-values were adjusted with the Benjamini-Hochberg method and the significance threshold of 0.05 FDR is indicated. Proteins located on chromosome 17p are indicated with a triangle. **f**: RNA, protein, and phosphoprotein levels of TP53 and FXR2 in samples with (n = 12) and without (n = 102) del17p. Boxplot shows median (middle line), 25th and 75th percentiles, whiskers extend to minimum and maximum excluding outliers (values greater than 1.5*IQR). Source data

For secondary genetic alterations, we mostly found proteins regulated in *cis*. Del(13q) comprises the known tumor suppressor genes *RB1* and *DIS3*, and their RNA and protein levels were consistently downregulated (Extended Data Fig. 5a,b). The most significantly downregulated protein was MYC binding protein 2 (MYCBP2), located on 13q (Extended Data Fig. 5a). MYCBP2 acts as an E3 ubiquitin ligase, playing a crucial role in modulating MYC transcriptional activity^31^. In patients with del(1p), we found downregulation of tumor suppressor and apoptosis regulator FAS-associated factor 1 (FAF1), as previously reported^26^ (Extended Data Fig. 5c,d). Deletion of 17p always comprises the tumor suppressor *TP53*, which was only detected in 18% of samples in our proteomic data. The most significantly downregulated protein in del(17p) cases was FXR2 (located 100 kb downstream of *TP53),* which is often codeleted with *TP53* in cancer (Extended Data Fig. 5e,f)^32^.

### The E2 ubiquitin ligase UBE2Q1 is a candidate oncoprotein in MM with 1q amplification

**Fig. 3:**
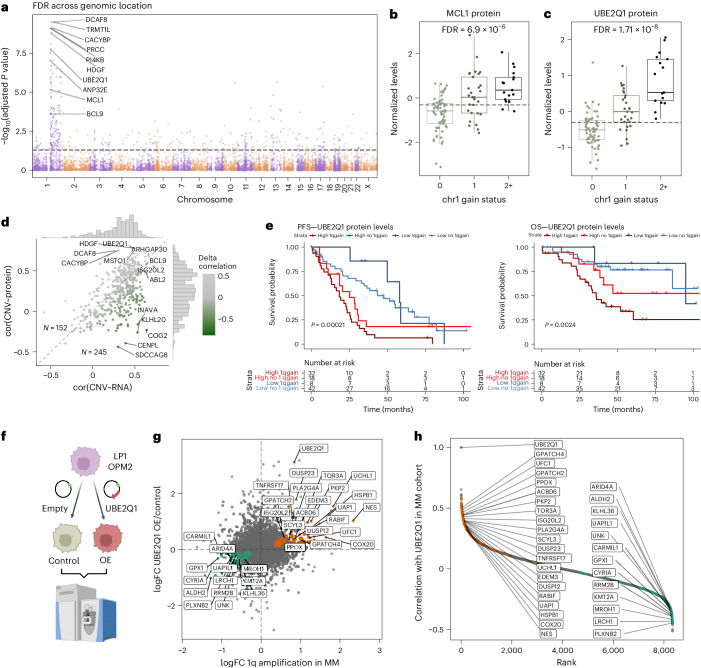
Identification of UBE2Q1 as a candidate protein for the aggressive phenotype of MM with gain/amp of chromosome 1q. **a**, Global protein levels in MM samples with 1q copy number gain (*n* = 46) were compared against all other samples (*n* = 68) with a two-sided, moderated two-sample *t*-test. The −log_10_(FDR) of each protein is plotted across genomic location. The significance threshold of 0.05 FDR is indicated. **b**, MCL1 protein levels in patients with MM grouped by 1q gain status. FDR for the comparison 1q gain versus no 1q gain is indicated (0: *n* = 68; 1: *n* = 29; 2+: *n* = 17). Box plots show median (middle line), 25th and 75th percentiles, whiskers extend to minimum and maximum excluding outliers (values greater than 1.5× IQR). **c**, UBE2Q1 protein levels in patients with MM grouped by 1q gain status. FDR for the comparison 1q gain versus no 1q gain is indicated (0: *n* = 68; 1: *n* = 29; 2+: *n* = 17). Box plot shows median (middle line), 25th and 75th percentiles, whiskers extend to minimum and maximum excluding outliers (values greater than 1.5× IQR). **d**, Genes located on chromosome 1q with at least ten valid value pairs in all datasets (RNA, DNA and protein) were extracted (*n* = 397 genes). The Pearson correlation coefficient of copy number determined by nanopore sequencing with RNA expression level (cor(CNV~RNA)) is plotted against the Pearson correlation coefficient of copy number with protein expression level (cor(CNV~protein). **e**, Kaplan–Meier plots show survival of patients grouped by UBE2Q1 protein levels (median) and 1q gain status. Survival in the different groups is compared by a log rank test. **f**, UBE2Q1 was overexpressed in LP1 and OPM2 cell lines. Empty vectors were used as a control. Cell lines were analyzed with label-free DIA proteomics (*n* = 4, biological replicates). **g**, Correlation of protein FCs in 1q gain myeloma patients (*x* axis) and UBE2Q1 overexpressing LP1 cells compared with control (*y* axis). Proteins regulated in LP1 cells (<0.05 FDR) and patients with MM with 1q gain (<0.1 FDR) and correlating with UBE2Q1 protein levels in myeloma cohort (*r* > 0.3 or *r* < −0.3) are indicated. **h**, Correlation analysis of UBE2Q1 with all other protein levels in newly diagnosed MM. Proteins are ranked by their Pearson correlation coefficient. The same proteins as in **g** are highlighted. Source data

**Extended Data Fig. 6:**
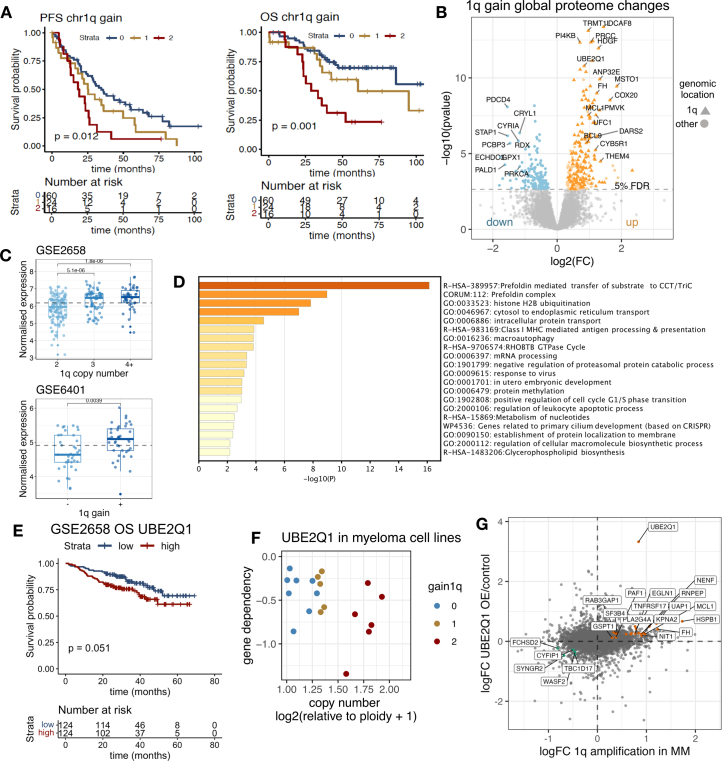
Influence of 1q amplifications on the proteome. **a**: Kaplan-Meier plot showing progression free and overall survival of myeloma patients stratified by chr1q gain (n = 100 patients). P-values were calculated with a log-rank test. **b**: Global protein levels in multiple myeloma samples with 1q copy number gain (n = 46) were compared against all other samples (n = 68) with a 2-sided, moderated 2-sample t-test. The log2 of fold change of each protein is plotted against its p-value. Proteins located on 1q are denoted with a triangle. P-values were adjusted with the Benjamini-Hochberg method and the significance threshold of 0.05 FDR is indicated. **c**: MCL1 RNA expression in multiple myeloma extracted from microrarray datasets GSE2658 (2: n = 134; 3: n = 70, 4 + : n = 44) and GSE6401 (1q gain n = 40, no 1q gain n = 37). Boxplot shows median (middle line), 25th and 75th percentiles, whiskers extend to minimum and maximum excluding outliers (values greater than 1.5 *IQR). The levels of MCL1 were compared by the two-sided t-test, p-values are indicated above the boxplots. P-values are adjusted using Bonferroni correction. **d**: Metascape GO term enrichment of proteins upregulated in 1q samples (< 0.05 FDR) that are not located on 1q. **e**: UBE2Q1 expression was extracted from Zhan et al. Microarray dataset (GSE2658). Kaplan-Meier plot shows overall survival of myeloma patients stratified by median UBE2Q1 expression. Survival in the groups is compared by the log rank test. **f**: Multiple myeloma cell line dependency data extracted from the depmap portal. The DNA copy number of UBE2Q1 is plotted against the genetic dependency. 1q copy number gains are indicated by color. **g**: Correlation of protein fold changes in 1q gain myeloma patients (x-axis) and UBE2Q1 overexpressing OPM2 compared to control (y-axis). Proteins regulated in OPM2 (<0.05 FDR) and in 1q patients (< 0.1 FDR) are indicated by color. Source data

Amplification of the long arm of chromosome 1 (+1q) is a well-established high-risk marker in MM and, consistent with previous studies, the number of 1q copies correlated with shorter overall survival (OS) in our cohort (Extended Data Fig. 6a)^6^. A large fraction of the upregulated proteins (147/237, 62%) is regulated in *cis* (on 1q), including many of the proteins previously suggested as potential oncogenic drivers such as ANP32E, BCL9 and MCL1 (Fig. 3a and Extended Data Fig. 6b)^33^. We observed only partial correlation of 1q status with protein levels of the clinical trial stage drug target MCL1 (Fig. 3b) and confirmed this finding with reanalysis of previously published expression data^34^ (Extended Data Fig. 6c). Several proteins involved in proteasomal degradation, proteostasis and protein folding pathways were upregulated in MM with 1q gain/amplification including proteins regulated in *cis* such as the E2 ligase UBE2Q1 (Fig. 3c) and the E3 ligase DCAF8 as well as in *trans* such as members of the chaperonin containing TCP-1 complex and E2 ligases UBE2G2 and UBE2H (Extended Data Fig. 6d). Although correlation of 1q genes from copy number (CN) to RNA and protein was in general high, many genes exhibited buffering effects of CNAs (Fig. 3d). The E2 ligase UBE2Q1 was the only 1q protein significantly associated with both adverse OS and progression-free survival (PFS) after false discovery rate (FDR) correction. The prognostic impact of UBE2Q1 protein expression was independent of 1q status, predicting outcomes even in patients without 1q chromosomal gain or amplification (Fig. 3e). Additionally, high RNA expression levels of UBE2Q1 were associated with shorter OS in an independent patient cohort^3^ (Extended Data Fig. 6e). Analysis of clustered regularly interspaced short palindromic repeat (CRISPR) KO screening data in MM cell lines revealed a correlation between UBE2Q1 genetic dependency and copy number status (Extended Data Fig. 6f). Given the role of UBE2Q1 in ubiquitination-mediated protein degradation, we evaluated the effect of UBE2Q1 overexpression in two MM cell lines (Fig. 3f). In UBE2Q1 overexpressing LP1 cells, we observed deregulation of proteins that also correlated with UBE2Q1 level expression in primary MM and were also differentially expressed in primary myeloma patients with 1q gain (Fig. 3g,h). These included the cell surface protein BCMA (TNFRSF17), ubiquitin hydrolase UCHL1, heat shock protein HSPB1, dual specificity phosphatases DUSP23 and DUSP12 and the stem cell marker nestin (NES). We also observed an overlap of regulated proteins in UBE2Q1 overexpressing OPM2 cells, although the effect was less pronounced (Extended Data Fig. 6g and Supplementary Table 8). These data imply that UBE2Q1, which is deregulated by DNA amplification of its gene, modulates protein levels of other proteins and points toward a role of UBE2Q1 in MM pathogenesis.

### Protein signatures in MGUS and PCL

**Fig. 4:**
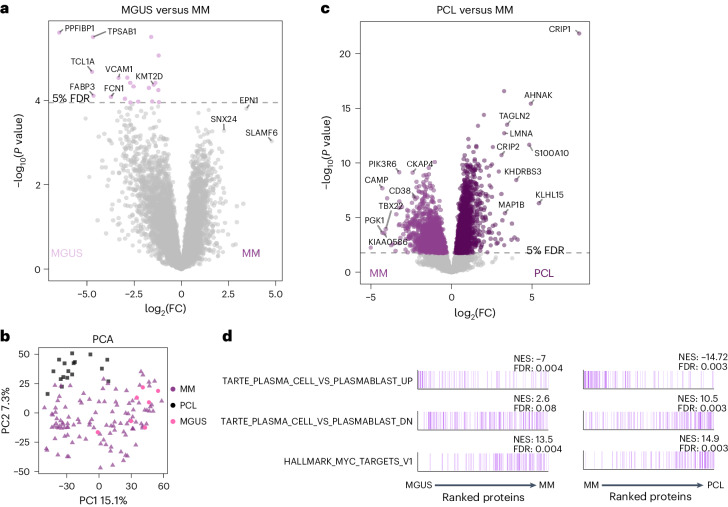
Proteome profiles of MGUS and PCL. **a**, Global protein levels in newly diagnosed MM samples (*n* = 114) were compared with those in premalignant MGUS samples (*n* = 7) with a two-sided, moderated two-sample *t*-test. The log_2_FC of each protein is plotted against its *P* value. *P* values were adjusted with the Benjamini–Hochberg method and the significance threshold of 0.05 FDR is indicated. **b**, PCA of global proteome data of newly diagnosed MM, MGUS and PCL samples. **c**, Global protein levels in MM samples (*n* = 114) were compared against PCL (*n* = 17) with a two-sided, moderated two-sample *t*-test. The log_2_ fold change of each protein is plotted against its *P* value. *P* values were adjusted with the Benjamini–Hochberg method and the significance threshold of 0.05 FDR is indicated. **d**, The mean log_2_ fold change of proteins in MM versus MGUS or PCL versus MM or samples was used as input for an ssGSEA analysis. The plot shows proteins ordered by their rank; proteins belonging to the respective gene set are marked by color. The normalized enrichment score (NES) and FDR of each gene set are indicated. Source data

**Extended Data Fig. 7:**
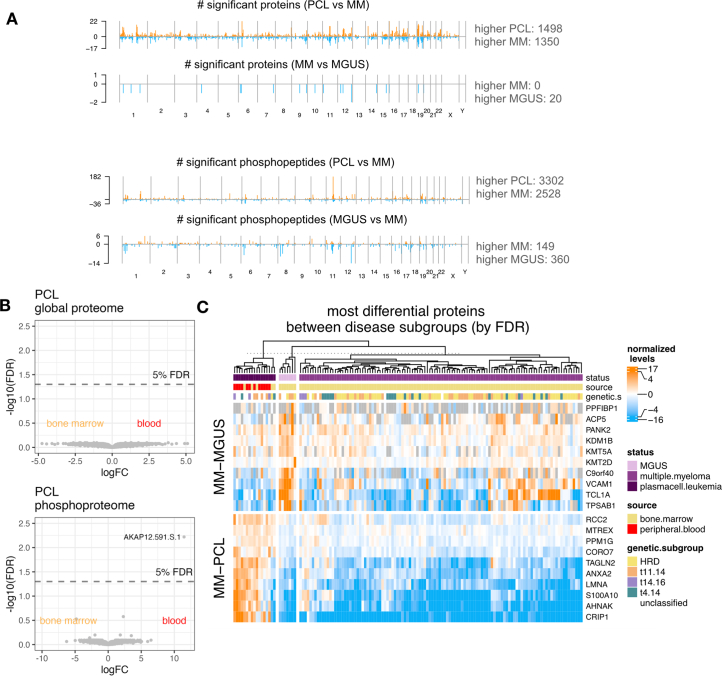
Differential protein levels in plasma cell leukemias indicate a highly proliferative phenotype. **a**: Global protein or phosphopeptide levels in multiple myeloma samples (n = 114) were compared against MGUS (n = 7) or plasma cell leukemia (n = 17) samples with a 2-sided, moderated 2-sample t-test. P-values were adjusted with the Benjamini-Hochberg method. Significant (< 0.05 FDR) proteins or phosphopeptides in each comparison are plotted across their genomic location. **b**: Global protein levels in plasma cell leukemia samples isolated from blood (n = 12) or bone marrow (n = 5) were compared with a 2-sided, moderated 2-sample t-test. The log2 of fold change of each protein is plotted against its p-value. P-values were adjusted with the Benjamini-Hochberg method and the significance threshold of 0.05 FDR is indicated. Bottom plot shows the same analysis for phosphoproteomic data. **c**: Heatmap displays normalized levels of the most significantly regulated proteins between MM and PCL or MM and MGUS (by FDR). Source data

MM develops from the premalignant state MGUS defined by the presence of less than 10% monoclonal plasma cells in the bone marrow and the absence of symptoms. Patients can remain in this state for >10 years without treatment. Proteomic analyses of seven MGUS cases revealed only a few differences to NDMM with deregulation of 20 proteins and 509 phosphopeptides (Fig. 4a and Extended Data Fig. 7a). Within the differentially expressed proteins, the histone methyltransferase KMT2D, a known tumor suppressor in B cell malignancies, was found at higher abundance in MGUS (Fig. 4a and Extended Data Fig. 7c)^35^.

PCL is a highly aggressive form of extramedullary myeloma with a poor outcome, where plasma cells acquire independence of the bone marrow microenvironment and enter the bloodstream. While genetically similar, the (phospho)proteome of PCL and MM differs significantly as demonstrated by principal component analysis (PCA) (Fig. 4b) and statistical comparison (Fig. 4c and Extended Data Fig. 7a), irrespective of whether the PCL cells were obtained from blood (*n* = 12) or bone marrow (*n* = 5) (Extended Data Fig. 7b,c). SsGSEA analysis revealed a gradual enrichment of proliferative and MYC target signatures from MGUS to MM to PCL (Fig. 4d). Among the most upregulated proteins in PCL are cysteine-rich protein 1 (CRIP1) and CRIP2, a protein also highly expressed in acute myeloid leukemia^36^. Further upregulated proteins in PCL include AHNAK, TAGLN2 and LMNA, which are linked to metastasis and aggressive disease in solid cancer (Extended Data Fig. 7c)^37^. Conversely, PCL cases displayed lower levels of the monoclonal antibody target CD38 (Fig. 4c).

### Proteomic-based outcome prediction

**Fig. 5:**
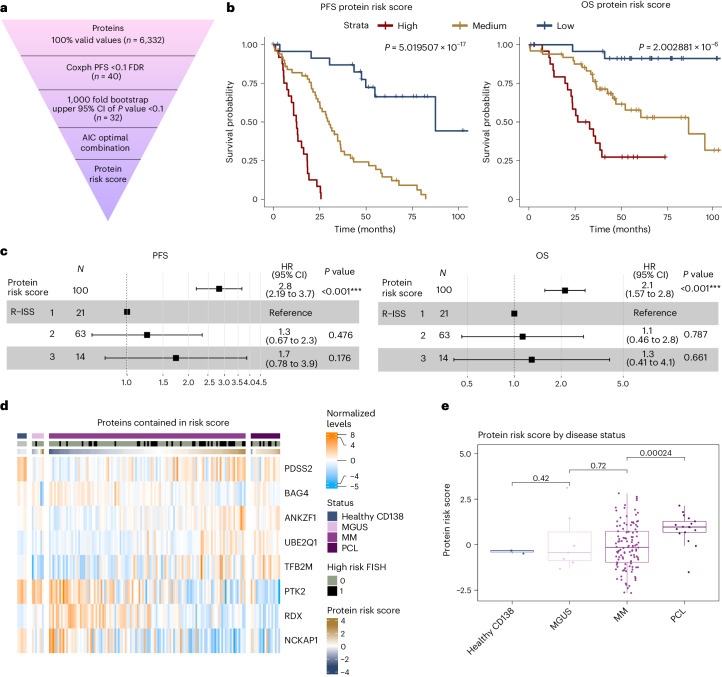
A proteomic risk score predicts outcome in NDMM. **a**, The workflow for the generation of a proteomic risk score in patients with NDMM who received a lenalidomide-based intensive treatment within clinical trials (*n* = 100). **b**, Kaplan–Meier plots show PFS and OS for patients according to the protein risk signature score divided by lowest quartile (low, *n* = 25), second and third quartile (medium, *n* = 50) and highest quartile (high, *n* = 25). Survival in the different groups is compared by the log rank test. **c**, Multivariable Cox regression analysis for PFS and OS including the protein risk score as continuous variable (hazard ratio (HR) per 1 point increase) and R-ISS. Data are represented as hazard ratio with 95% confidence interval (CI). Significance was tested with a Wald test. **d**, Expression of proteins contained in the protein high-risk score across samples from healthy donors, patients with premalignancy MGUS, MM and PCL. **e**, Protein risk score values calculated for the proteome data of healthy plasma cells, MGUS, MM and PCL samples. *P* values from a two-sided Student’s *t*-test are indicated. Healthy CD138: *n* = 3; MGUS: *n* = 7; MM: *n* = 114; PCL: *n* = 17. Box plots show median (middle line), 25th and 75th percentiles, whiskers extend to minimum and maximum excluding outliers (values greater than 1.5× IQR). Source data

**Extended Data Fig. 8:**
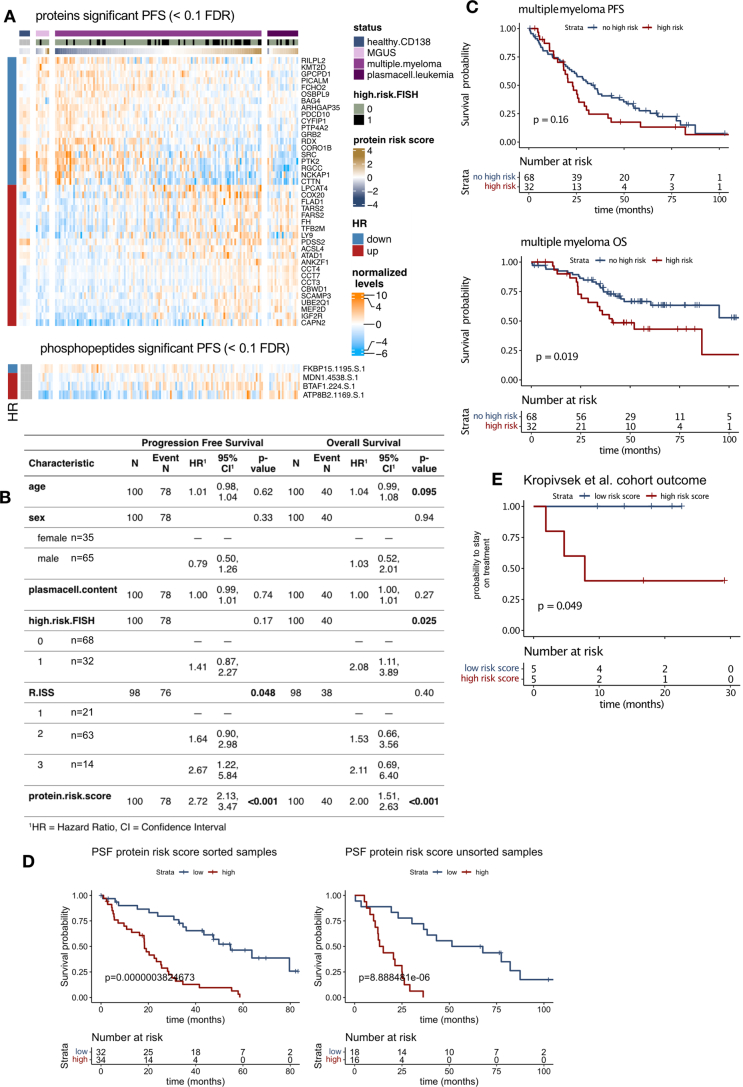
Proteins and phosphopeptides associated with outcome. **a**: Fully quantified proteins and phosphopeptides were investigated for their correlation with progression-free survival with a univariate Cox regression analysis as a continuous variable. The resulting p-values were subjected to multiple testing control with Benjamini-Hochberg. Normalized expression levels of proteins and phosphopeptides passing the 0.1 FDR cutoff are plotted as a heatmap. Row annotation indicates hazard ratios > 1 (up) or < 1 (down). **b**: Table showing the impact of clinical parameters and protein risk score on progression-free survival (PFS) and overall survival (OS). P-values were calculated with univariate Cox regression analysis. **c**: Kaplan-Maier plots showing PFS and OS curves of patients with (blue) and without (red) at least one high-risk FISH marker (del(17p), t(4;14), +1q21). P-values were calculated with a log-rank test. Patients treated within the DSMM clinical trials that received a lenalidomide-based induction therapy followed by high-dose melphalan/autologous hematopoietic stem cell transplantation and lenalidomide maintenance were included (n = 100) in the survival analysis. **d**: Kaplan-Meier plots showing progression-free survival (PFS) for patients according to the protein risk signature score in samples with and without CD138 MACS sorting. Survival in the groups is compared by the log rank test. **e**: Proteomics data was extracted from Kropvisek et al. and protein risk score was calculated for untreated myeloma patients (n = 10). Kaplan-Meier plot shows time to the next treatment or death for myeloma patients stratified by median risk score. Survival in the groups is compared by the log rank test. Source data

Risk stratification of NDMM is currently based on R-ISS and in our cohort we concordantly observed a significant impact of R-ISS on survival while other parameters had no effect (Extended Data Fig. 8b,c). We evaluated whether proteomics and phosphoproteomics provide prognostic information in addition to R-ISS, the current standard for risk stratification in MM. We conducted single-variable Cox regression analysis on PFS and OS using fully quantified proteins and phosphopeptides in 100 patients treated in the Deutsche Studiengruppe Multiples Myelom (DSMM) XII, XIII and XIV clinical trials. Despite variations in induction therapy, all patients were scheduled to receive a lenalidomide-based induction, high-dosage melphalan with autologous stem cell transplantation (auto-SCT) and lenalidomide maintenance therapy (for details, see Methods). In total, 40 proteins and 4 phosphopeptides had FDR <0.1 and one protein FDR <0.05 (Supplementary Table 9 and Extended Data Fig. 8a). Applying a bootstrapping approach and model optimization (Fig. 5a), we defined a protein risk score containing protein level information of eight proteins with differing weights, including the 1q protein UBE2Q1 (Supplementary Table 9). Patients with a high protein risk score (*n* = 25) had a median PFS of 12.5 months as compared with 30.0 months in patients with a median score (*n* = 50), and 87.4 months in patients with a low score (*n* = 25), which translated to a median OS of 29.6, 86.3 and 108.1 months, respectively (Fig. 5b). The prognostic value of the protein risk score remained consistent across CD138-enriched and nonenriched samples (Extended Data Fig. 8d) and was independent of R-ISS (Fig. 5c). Strikingly, the protein risk score gradually increased following disease aggressiveness from MGUS (median score −0.43) to NDMM (median score −0.15) and PCL (median score 0.97) (Fig. 5d,e). The proteomic risk signature had a significant impact on outcome in an independent, external cohort of patients with NDMM recently published by Kropivsek et al.^21^ despite the small number of patients as well as differences in treatment and proteomic data acquisition (Extended Data Fig. 8e).

### Identification of MM-selective and essential proteins

**Fig. 6:**
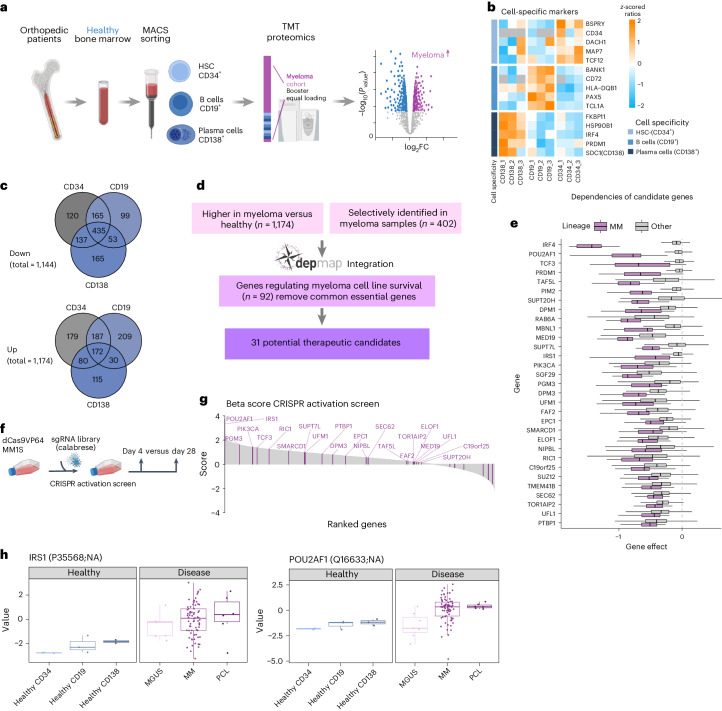
Integrated proteomic and genetic screens reveal drivers of MM cell growth. **a**, Hematopoietic cell populations were sorted using MACS enrichment for the surface markers CD34 (hematopoietic stem and progenitor cells (HSCs)), CD19 (B cells) and CD138 (plasma cells) from bone marrow of individuals without hematologic malignancy (*n* = 3). Proteins were quantified via TMT with a booster channel approach. Booster and equal loading control were identical to the internal standard used for TMT analysis of cohort samples. **b**, Protein levels of cell lineage-specific markers in healthy samples. *z*-scored TMT ratios are displayed. **c**, Proteins in MACS sorted healthy bone marrow and CD138^+^ sorted MM samples were compared with a two-sided, moderated two-sample *t*-test. *P* values were adjusted with the Benjamini–Hochberg method. The total number of regulated proteins is indicated, the Venn diagrams show overlap of up- and downregulated proteins in MM samples compared with healthy samples (FDR < 0.1). **d**, Data analysis workflow to identify potential therapeutic candidates from myeloma upregulated or specifically expressed proteins. **e**, Gene dependency scores from CRISPR–Cas9 KO screening data from the depmap portal. The gene effect of potential therapeutic targets in myeloma (*n* = 18) and other cell lines (*n* = 1,082) is displayed. The RNA to protein correlation in myeloma cohort is indicated for each candidate gene. Box plot shows median (middle line), 25th and 75th percentiles, whiskers extend to minimum and maximum excluding outliers (values greater than 1.5× IQR). **f**, The workflow for a genome-wide CRISPR–Cas9 activation screen using the Calabrese library performed in the MM cell line MM.1S. **g**, Gene effect on proliferation ranked by beta score. A higher beta score indicates expansion of cells carrying the indicated sgRNAs. The MAGeCK MLE algorithm was applied for the analysis of beta scores and *P* values. Potential targets identified by proteomic analysis are marked in purple. **h**, Protein levels of IRS1 and POU2AF1 across healthy and malignant cell populations. Healthy CD138: *n* = 3; healthy CD19: *n* = 3, healthy CD34: *n* = 3; MGUS: *n* = 7; MM: *n* = 114; PCL: *n* = 17. Box plot shows median (middle line), 25th and 75th percentiles, whiskers extend to minimum and maximum excluding outliers (values greater than 1.5× IQR). Source data

**Extended Data Fig. 9:**
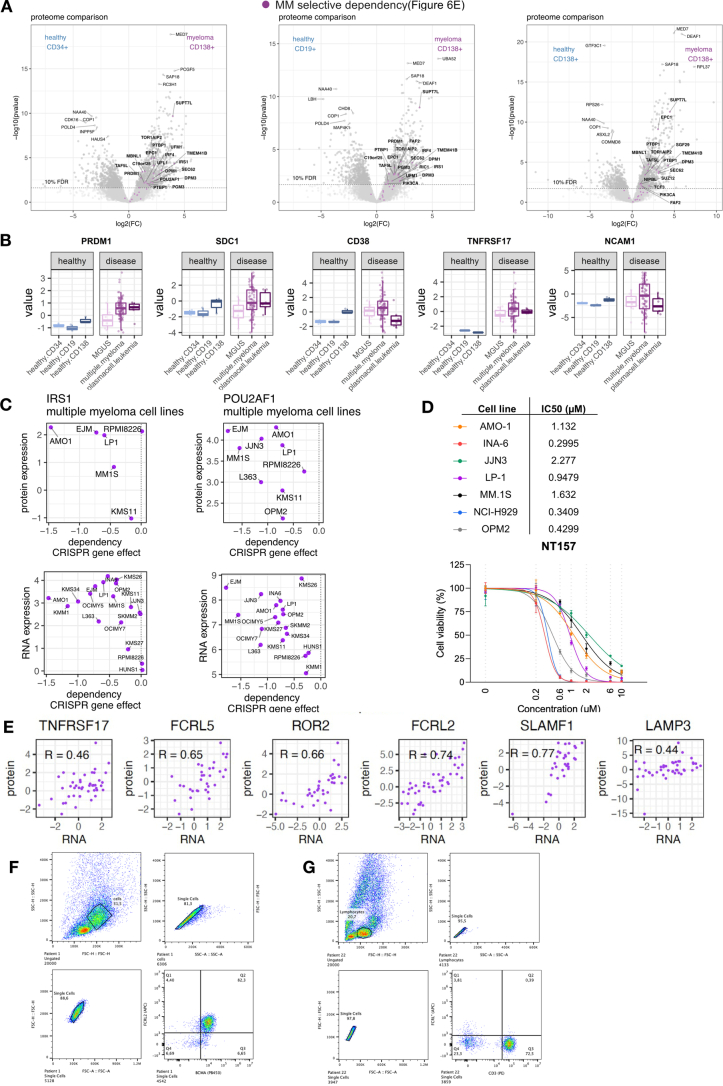
Comparison of multiple myeloma with healthy bone marrow reveals potential therapeutic targets. **a**: Global protein levels of multiple myeloma samples (MACS sorted samples only, n = 76) and healthy bone marrow cells sorted for CD138+ (plasma cells, n = 3), CD19+ (B cells, n = 3) and CD34+ (HSC, n = 3) were compared with a 2-sided, moderated 2-sample t-test. The log2 of fold change of each protein is plotted against its p-value. P-values were adjusted with the Benjamini-Hochberg method and the significance threshold of 0.1 FDR is indicated. Data was integrated with the depmap database and potential therapeutic targets (Fig. 6d) are indicated as purple stars. **b**: Protein levels of selected plasma cell-specific proteins in healthy and disease samples. Healthy CD138: n = 3; healthy CD19: n = 3, healthy CD34: n = 3; MGUS: n = 7; MM: n = 114;. PLC: n = 17. Boxplot shows median (middle line), 25th and 75th percentiles, whiskers extend to minimum and maximum excluding outliers (values greater than 1.5 *IQR). **c**: Protein (top) or RNA (bottom) expression of IRS1 and POU2AF1 in multiple myeloma cell lines plotted against genetic dependency. Data was extracted from the depmap database and Goncalves et al. **d**: Cell viability of multiple myeloma cell lines treated for 96 h with the IRS1 inhibitor NT157 in biological triplicates. Concentration is indicated in µM. Data is represented as mean ± standard deviation. **e**: RNA to protein correlation of selected surface markers in myeloma samples displayed in Fig. 7. **f** and **g**: Representative plot showing gating strategy for the FACS analysis in Fig. 7d–f: Multiple myeloma cells, G: non-malignant cells on example of T cells. Source data

We utilized TMT-based proteomics with a booster channel to identify proteins specific to MM cells compared with hematopoietic stem and progenitor cells (CD34^+^), B cells (CD19^+^) and plasma cells (CD138^+^) isolated from the bone marrow of healthy donors (Fig. 6a). Key hematopoietic lineage markers behaved as expected with higher levels of PAX5 in B cells, CD34 in stem/progenitor cells and IRF4 in CD138^+^ plasma cells (Fig. 6b). A comparison of MM cells with each of the three healthy populations revealed 1,475, 1,350 and 1,187 significantly regulated proteins (FDR <0.1) in MM as compared with CD34^+^, CD19^+^ and CD138^+^ healthy cells, respectively (Fig. 6c and Extended Data Fig. 9a). Among the proteins consistently upregulated in MM were ribosomal proteins and heat shock proteins (Extended Data Fig. 9a). Several markers of plasma cell differentiation including PRDM1, CD56 (NCAM1) and BCMA (TNFRSF17) were higher expressed in MM cells while for CD138 (SDC1) and CD38 no major differences were observed (Extended Data Fig. 9b). We combined the list of significantly upregulated proteins in any of the three comparisons (Fig. 6c) with proteins selectively identified in myeloma cells (402 proteins) and performed integrated analysis with genetic dependency data (depmap.org)^38^ (Fig. 6d). To detect myeloma-specific vulnerabilities, genes were filtered by their median dependency in myeloma versus nonmyeloma cell lines applying a cutoff based on the lenalidomide targets IKZF1 and IKZF3 (refs. ^39,40^). This resulted in a candidate target list of 31 proteins that included known MM survival factors such as transcription factors IRF4 and PRDM1 and kinases PIM2 and PIK3CA (Fig. 6e)^41^. Among the proteins not previously linked to MM were three members (TAF5L, SUPT7L and SUPT20H) of the SAGA complex, a posttranslational regulator of MYC transcriptional activity that is important for myeloma growth. Two additional SAGA subunits, SUPT3H and TAF12, were also upregulated in MM but did not pass the filter for selective dependency^42^. The candidate list further included members of the dolichol-phosphate mannose synthase complex DPM1 and DPM3 and the ubiquitin-like modifier UFM1 as well as its ligase UFL1. To further evaluate the role of proteins in MM, we performed a complementary whole-genome CRISPR activation screen in the MM.1S cell line (Fig. 6f and Supplementary Table 10). Strikingly, the top genes driving MM cell growth were POU2AF1 and IRS1, two proteins highly expressed and essential for MM (Fig. 6g,h). *POU2AF1*, encoding the OCA-B transcriptional coactivator, is a B cell differentiation factor essential for germinal center formation and several B cell neoplasias, including lymphoma^43^ and MM^44^. IRS1 is a downstream signaling protein of insulin growth factor 1 receptor (IGF1R) and is highly phosphorylated in MM cells when IGF1 binds to IGF1R^45^. Expression of IRS1 and POU2AF1 in MM cell lines extracted from the Cancer Cell Line Encyclopedia and the pan cancer proteomic map^46^ is highly correlated with genetic dependency (Extended Data Fig. 9c). Treatment with the IRS1 inhibitor NT157 (ref. ^47^) reduced proliferation in MM cell lines, highlighting IRS1 as a potential selective target for therapy (Extended Data Fig. 9d). In aggregate, these data demonstrate that integrated proteomic analysis in primary patient cells with functional genetics in cell lines reveals potential therapeutic vulnerabilities in MM.

### Proteomics reveals candidates for immunotherapies

**Fig. 7:**
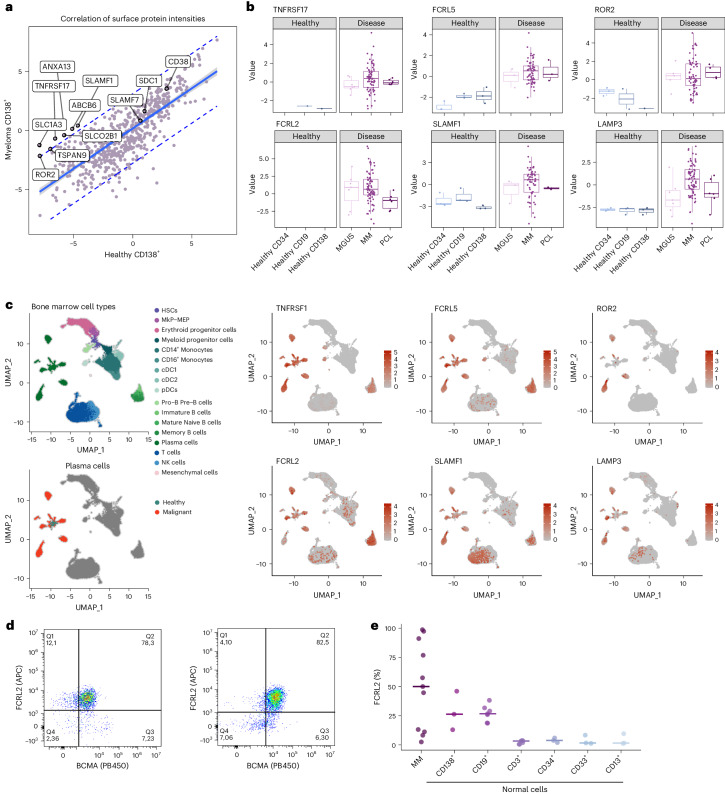
Identification of surface proteins on MM cells. **a**, The identified surface proteins from the healthy to disease comparison were extracted by integrating proteomics data with the cancer surfaceome atlas. The plot shows the correlation of median-normalized raw intensities of surface proteins in CD138^+^ sorted MM and healthy bone marrow samples. The 95% confidence interval is indicated with a blue line, the 95% prediction interval is indicated with dashed blue lines. **b**, Protein levels of selected surface proteins in healthy hematopoietic cells and malignant plasma cells. Healthy CD138: *n* = 3; healthy CD19: *n* = 3, healthy CD34: *n* = 3; MGUS: *n* = 7; MM: *n* = 114; PCL: *n* = 17. Box plot shows median (middle line), 25th and 75th percentiles, whiskers extend to minimum and maximum excluding outliers (values greater than 1.5× IQR). **c**, UMAP plots showing single-cell RNA sequencing data of bone marrow from healthy and patients with MM. Cells are colored by cell type, malignancy status or by normalized RNA expression levels of selected surface proteins. **d**, FACS analysis of BCMA (TNFRSF17) (*x* axis) and FCRL2 (*y* axis) expression in MM samples. Two representative examples of patients with MM with high FCRL2 expression were selected. **e**, The percentage of FCRL2-positive cells in MM cells and minimal to no expression in other normal hematologic cell populations, *n* = 19. MkP, megakaryocyte progenitor; MAP, megakaryocyte/erythrocyte progenitor; DC, dendritic cell; NK, natural killer. Source data

Immunotherapies such as CAR-T cells and bispecific antibodies targeting BCMA and GPCR5D are approved and highly effective treatments for MM^48,49^. To identify additional MM selective cell surface proteins, we integrated our comparison of healthy and malignant plasma cells with the cancer surface proteome resource^50^ (Fig. 7a). While TNFRSF17 (BCMA) was highly specific for myeloma samples, other immunotherapy targets such as CD38, CD138 (SDC1) and SLAMF7 were not or only moderately higher expressed in MM versus healthy plasma cells. In addition, we detected several proteins with expression levels higher in MM cells, including Fc receptor-like 2 and 5 (FCRL2 and 5), receptor tyrosine kinase like orphan receptor 2 (ROR2), signaling lymphocytic activation molecule family member 1 (SLAMF1) and lysosomal associated membrane protein 3 (LAMP3) (Fig. 7b). All proteins displayed good RNA-to-protein correlation in our dataset (Extended Data Fig. 9e) and evaluation of these targets in single-cell RNA sequencing data^51^ further confirmed their selective and higher expression in malignant plasma cells (Fig. 7c). FCRL5 is currently being explored as an immunotherapy target in MM in clinical trials^52^. Leveraging single-cell RNA sequencing data from the protein atlas (https://www.proteinatlas.org/)^53^ revealed ROR2, LAMP3 and SLAMF1 to be expressed in non-hematopoietic tissue and we thus chose to further evaluate FCRL2, which is only expressed on plasma and B cells. Flow cytometry in primary patient and healthy donor bone marrow confirmed FCRL2 surface expression on MM cells in 7 of 11 patients and showed moderate or low expression on healthy plasma and B cells and other hematopoietic cells, respectively (Fig. 7d,e and Extended Data Fig. 9f,g).

## Discussion

We provide a proteogenomic landscape of newly diagnosed, untreated MM, covering the major cytogenetic alterations of this disease. Including comparisons with healthy cells, MGUS and PCL, and correlation with clinical data, MM-specific proteins can be explored in the context of disease progression. Analysis of >100 well-annotated primary patient samples and integration with DNA and RNA sequencing allowed us to map the consequences of recurrent genetic alterations to the (phospho)proteome. A low correlation of RNA to protein levels was observed in myeloma cells, consistent with proteogenomic studies in other types of hematologic and solid cancer^9–11,13^. This was especially true for the proteins involved in protein homeostasis, such as proteasome formation, ubiquitination and splicing. In contrast, RNA and protein levels of regulators of B cell differentiation, IgH translocation partners and those encoded in CNAs showed higher correlation. Multiple genetic alterations affecting cell cycle regulation, including cyclin D translocations or upregulation of RB1 phosphorylation as well as *RB1* deletions, had a major impact on the (phospho)proteome, highlighting cell cycle dysregulation as a hallmark of MM. In patients with primary IgH translocations, the recurrent translocation partners were, in general, the most upregulated proteins and RNA transcripts, with the exception of FGFR3, which was only elevated in a subset of cases with t(4;14). FGFR3 protein abundance independent of t(4;14) was predictive of downstream signaling and sensitivity to FGFR3 inhibition. The pronounced deregulation of proteins involved in the apoptosis pathway and B cell markers observed in t(11;14) myeloma provides a possible link to the enhanced sensitivity of these cases to BCL2 inhibition^22^. These findings may guide future studies to find more reliable predictive protein-based biomarkers for personalized treatment in MM. In line with this, proteomic-based prediction for ex vivo drug sensitivity in primary MM cells has recently been demonstrated by Kropivsek et al.^21^.

Amplification of chromosome 1q is an established high-risk marker in MM and also other types of cancer. However, which of the proteins encoded on 1q confer therapy resistance is not completely understood^6^. While several previously described 1q candidates such as ANP32E, BCL9 and MCL1 were found upregulated on the protein level in +1q cases, our integrated analysis identified the E2 ubiquitin ligase UBE2Q1 as a 1q protein highly correlated with outcome. Consistent with our findings in MM, high UBE2Q1 expression levels are associated with shorter survival in other cancers indicating a tumor-agnostic role in conferring therapy resistance^54–56^. E2 ubiquitin ligases, which as enzymes are in principle amenable for pharmacologic intervention, mediate ubiquitin transfer to a substrate protein via an E3 ligase and thus can regulate their substrate proteins on the posttranslational level. Consistently, we show that UBE2Q1 regulates many of the proteins also found differentially expressed in patient samples with 1q gain. The E3 ubiquitin ligase(s) for UBE2Q1 as well as its substrates implicated in drug resistance are currently not known and warrant further studies. In addition to UBE2Q1, we found other members of the ubiquitin-proteasome system deregulated either directly by chromosomal events in *cis* or through *trans* effects including E3 ligases DCAF8 (Chr 1q) and MYCBP2 (Chr 13q), the deubiquitinating enzymes UCHL1 and USP4 (Chr 3) and ubiquitin-like modifiers UFL1 and UFM1. Conceivably, altered levels of these enzymes lead to posttranslational regulation of their substrates, which to some extent may explain the low RNA–protein correlation observed in MM.

Outcome prediction is of high clinical relevance in cancer to identify patients with aggressive disease and to personalize therapy. We identified a protein risk signature that was highly predictive for outcome and independent of the R-ISS in patients with NDMM from three consecutive DSMM clinical trials that incorporated the major therapy principles still included in current first-line therapies. The proteins in the risk signature, which include the 1q protein UBE2Q1, are not associated with known drug mechanisms and do not overlap with genes from RNA-based risk signatures such as GEP70 or SKY92, highlighting additional value provided by proteomics^4,57^. Furthermore, our protein risk signature was associated with disease stage and could be validated in an independent cohort^21^ that was treated with different treatment modalities, further implying that these proteins are associated with aggressive disease. These findings need to be evaluated in larger patient cohorts in the context of currently applied therapy regimens to determine clinical applicability. While we could only investigate a small but representative subset of patients of the DSMM trials due to sample availability, technically, (phospho)proteomics could be performed for the majority of myeloma patients, similar to cytogenetic and RNA expression analysis. Since the reliability of global analyses of bulk tumor samples in general depends on tumor cell purity, we show that our results including outcome and conclusions are robust and independent of sorting status if a cutoff of 75% tumor cell purity is applied.

We found the premalignancy MGUS and symptomatic MM to be almost indistinguishable on the proteome level, which in part may be explained by the strong impact of genetic alterations that are already present in MGUS^35^. In contrast, PCLs have a more distinct protein expression profile and we observed some overlap of upregulated proteins to other disseminated, aggressive malignancies such as acute leukemia^36^.

Proteomic profiling of healthy and malignant plasma cells and integration with CRISPR dependency data revealed myeloma-specific vulnerabilities. Besides well-established B cell differentiation regulators, including IRF4 and PRDM1, we found the transcriptional coactivator POU2AF1 (OCA-B, BOB1) as a highly expressed protein and essential in MM. These findings are in line with a recent study describing POU2AF1 as a regulator of genes important for MM proliferation^38^. We detected insulin response substrate-1 (IRS1) as another potential drug target in MM. Insulin growth factor signaling leads to the activation of AKT, which has been shown to promote myeloma growth^45^ but is also important for other tissues. Our data indicate that IRS1 as compared with IGF-R1 is a highly selective target of this pathway in MM cells. Our integrated analyses further point to a potential role of the SAGA complex in MM that, among other functions, is a posttranslational regulator of MYC, providing a potential link to the transformation of plasma cells^58^.

T cell therapies such as CAR-T and bispecific antibodies are revolutionizing MM treatment, showing remarkable effectiveness in multidrug resistant patients. However, resistance can arise from loss or mutation of targeted surface proteins, as shown for BCMA and GPCR5D^59,60^, highlighting the need for additional targets. Our combined proteomic and single-cell RNA sequencing approach reliably detected MM-specific surface proteins, including candidates recently found by an orthogonal approach using surface biotinylation or glycoprotein capture^61,62^. We validated FCRL2 as a surface protein on primary MM cells. FCRL2 is also expressed on chronic lymphocytic leukemia cells^63^ and may be exploited as a potential immunotherapy target in myeloma and other B cell malignancies.

Collectively, the proteomic landscape of plasma cell malignancies described here provides a powerful resource that can easily be assessed through an online tool for interactive self-exploration (https://myelomaprot.mdc-berlin.de) to promote research on MM biology, risk stratification and novel therapies.

## Methods

### Study cohort

A total of 138 patients were included in the proteomics study (114 NDMM, 17 PCL and 7 MGUS cases). Inclusion criteria were the availability of myeloma cells of appropriate quantity and quality for proteomic and genetic analyses and available information on FISH-bases cytogenetics and clinical parameters. All patients provided written informed consent according to the Declaration of Helsinki. The study was approved by the responsible ethic committees Ulm University (136/20, 307/08) and Charité Universitätsmedizin Berlin (EA2/142/20). Clinical trials of the DSMM and sample collection were approved by the ethics committee of Würzburg University (2008-000007-28, 145-11).

Patient characteristics are summarized in Supplementary Table 1. One hundred out of 114 newly diagnosed patients were treated within one of the DSMM XII–XIV clinical trial and had available outcome data (NCT00925821, NCT01090089 and NCT01685814)^64^. All of these 100 patients were scheduled to receive a lenalidomide-based intensive therapy within a clinical trial.

DSMM XII/NCT00925821 (*N* = 12): induction therapy with four cycles lenalidomide/adriamycine/dexamethasone. All patients were scheduled to receive high-dose melphalan/auto-SCT, while the nature of the second SCT was determined by risk stratification: high-risk patients (cytogenetics, ISS) were scheduled to undergo allogeneic stem cell transplantation (*N* = 3) followed by lenalidomide maintenance while standard-risk patients received a second auto-SCT followed by lenalidomide maintenance for 1 year^64^.

DSMM XIII, arm A2/NCT01090089 (*N* = 20): induction therapy with three cycles of lenalidomide/dexamethasone followed by two cycles of high-dose melphalan/auto-SCT and lenalidomide maintenance until progression.

DSMM XIV NCT01685814 (*N* = 68): induction therapy randomized between four cycles lenalidomide/adriamycine/dexamethasone (*N* = 36) or three cycles lenalidomide/bortezomib/dexamethasone (*N* = 32) followed by high-dose melphalan/auto-SCT, second randomization in patients with very good partial response (VGPR) or better directly to lenalidomide maintenance until progression or a second cycle of high-dose melphalan/auto-SCT followed by lenalidomide maintenance for 3 years. Patients not achieving VGPR after the first high-dose melphalan/auto-SCT were randomized to receive a second cycle of high-dose melphalan/auto-SCT followed by lenalidomide maintenance for 3 years or allogeneic stem cell transplantation (*N* = 3) followed by 1 year of lenalidomide maintenance.

No significant difference was observed for PFS and OS across the patients treated in the three different trials.

Healthy control cells were obtained from orthopedic surgery patients without evidence for malignancy. The median age of the healthy donors was 63 years (range, 57–78 years). All donors provided written informed consent according to the Declaration of Helsinki and the study was approved by the responsible ethic committee Charité – Universitätsmedizin Berlin (EA4/115/21).

### Cell isolation

Except for 12 PCL samples from peripheral blood, all samples were collected from bone marrow aspiration. Mononuclear cells were isolated with a Ficoll gradient and plasma cell content was determined morphologically. The majority of samples (89/138) were enriched for CD138^+^ cells via magnetic-activated cell sorting (MACS) directly after mononuclear cell isolation using magnetic beads conjugated to a human CD138-specific antibody (130-051-301, Miltenyi). Non-MACS enriched samples (49/138) were selected for a plasma cell content >75% and had an average CD138^+^ purity of 85%. Healthy bone marrow mononuclear cells were isolated by Ficoll gradient and CD34^+^, CD19^+^ and CD138^+^ cells were enriched with MACS antibody bead conjugates (all Miltenyi), according to the manufacturer’s protocol. For each cell population of healthy bone marrow cells, three replicates were obtained. Replicates one and two were obtained from separate individuals and replicate three was obtained by pooling material from three different donors due to limitations in sample material.

### FISH analysis

FISH in combination with immunofluorescent detection of light chain-restricted plasma cells was performed on plasma cells from patients. Genetic regions of interest for the diagnosis of MM and their translocation partners were detected. FISH was performed according to standardized protocols using commercially available probes (Abbott Laboratories and MetaSystems).

### DNA preparation and nanopore sequencing

DNA was isolated with the AllPrep DNA/RNA kit (QIAGEN, 80204). RNA and DNA were extracted from the same sample while protein was extracted from a different aliquot of the same patient/time point sample. Nanopore DNA sequencing was performed with the Oxford Nanopore Technologies (ONT) platform. Libraries containing either a pool of three samples or just a single sample were prepped with the Rapid Barcoding Sequencing kit (SQK-RBK004, ONT) using approximately 350 ng starting material for each sample in a pool of three or 400 ng of starting material for a single run (Rapid Sequencing kit, SQK RAD004). A maximum amount of 850 ng library was loaded onto the flow cell (FLO-MIN106D, R 9.4.1, ONT) and sequenced on a GridION sequencer (ONT), according to the manufacturer’s instructions.

### RNA sequencing library preparation and sequencing

RNA was isolated with the AllPrep DNA/RNA kit (QIAGEN, 80204). Library preparation was performed from 20 to 100 ng of input total RNA per sample using the TruSeq Stranded Exome RNA kit (Illumina), according to the manufacturer’s instructions. The pooled RNA libraries were sequenced on an Illumina HiSeq2000 with 50-bp single-end reads with an average coverage of 36.6 × 10^6^ reads per sample.

### Protein extraction and digestion

Samples were lysed at 4 °C with urea lysis buffer as previously described^65^. Protein lysates were reduced with 5 mM dithiothreitol for 1 h and alkylated with 10 mM iodoacetamide for 45 min in the dark. Samples were subsequently diluted 1:4 with 50 mM Tris–HCl, pH 8 and sequencing grade LysC (Wako Chemicals) was added at a weight-to-weight ratio of 1:50. After 2 h, sequencing grade trypsin (Promega) was added at a weight-to-weight ratio of 1:50 and digestion was completed overnight. Samples were acidified with formic acid and centrifuged to remove precipitated material (20,000*g*, 15 min). The supernatant was desalted with Sep-Pak C18 cc Cartridges (Waters).

### TMTpro labeling of peptides

Desalted and dried peptides were labeled with TMTpro 16 plex reagents (Thermo Scientific) according to the manufacturer’s instructions and at a sample-to-tag ratio of 1:7 (*w*/*w*). After confirming successful labeling, TMT-labeled peptides of cohort samples were randomly combined into ten TMTpro plexes (see Supplementary Table 11 for TMT channel allocation). For TMT plex 1–9, 75 µg peptides per channel were used and 45 µg of peptides per channel were used for TMT plex 10. An equal loading internal standard that consisted of a mix of all cohort samples was included in each TMT plex. Samples from healthy bone marrow donors were analyzed in an 11th TMTpro plex with 10 µg peptides per sample and an equal loading internal standard that was the same as for the cohort samples. The 11th TMT plex also contained a booster channel (500 µg peptides) that was identical to the internal standard and the two TMT channels next to it were left empty to prevent signal spillover. Combined TMT samples were dried down and resuspended in liquid chromatography sample buffer (3% acetonitrile (ACN), 0.1% formic acid) before desalting with Sep-Pak C18 cc Cartridges (Waters).

### Peptide fractionation of TMT-labeled samples

Dried TMT-labeled samples were resuspended in high pH buffer A (5 mM ammonium formate, 2% ACN) before offline high pH reverse phase fractionation by high-performance liquid chromatography (HPLC) on an UltiMate 3000 HPLC (Thermo Scientific) with an XBridge Peptide BEH C18 (130 A˚, 3.5 µm; 4.6 mm × 250 mm) column (Waters) as previously described (Mertins et al.^65^). Each fractionated TMT plex was pooled into 24 or 28 fractions and 10% of each fraction was reserved for global proteome measurements. The remaining fractions were further pooled into 12 or 14 fractions per TMT plex for phosphoproteomics. Dried global proteome fractions or immobilized metal affinity chromatography-enriched phosphopeptides were reconstituted in liquid chromatography sample buffer before mass spectrometric measurements.

### Phosphopeptide enrichment

Phosphopeptide enrichment was performed with immobilized metal affinity chromatography automated on an AssayMap Bravo System (Agilent) equipped with AssayMAP Fe(III)-NTA cartridges.

### Liquid chromatography–mass spectrometry

Samples were fractionated online with a 25-cm column packed in-house with C18-AQ 1.9 µm beads (Dr. Maisch Reprosil-Pur 120). Samples were separated with a gradient of mobile phase A (0.1% formic acid and 3% acetonitrile in water) and mobile phase B (0.1% formic acid, 90% acetonitrile in water) at a flow rate of 250 µl min^−1^. TMT samples were separated with an EASY nLC 1200 HPLC system and temperature of the column was controlled by a column oven set to 45 °C. For a 2 h gradient, mobile phase B was increased from 4% to 30% in the first 88 min, followed by an increase to 60% B in 10 min and a plateau of 90% B for 5 min, followed by 50% buffer B for 5 min. For a 4 h gradient, mobile phase B was increased from 3% to 30% in the first 192 min followed by an increase to 60% B in 10 min, a plateau of 90% B for 5 min and 5 min 50% buffer B. All TMT fractions were measured with a 2 h gradient. To boost identification in the 11th TMT plex with healthy bone marrow samples, fractions of plex 11 were additionally measured with a 4 h gradient. MS data of TMT samples was acquired in profile centroid mode and data-dependent acquisition on a Q Exactive HF-X (Thermo Fisher). MS1 scans were acquired at 60,000 resolution, scan range of 350–1,500 *m*/*z*, maximum injection time (IT) of 10 ms and automatic gain control (AGC) target value of 3e6. The 20 most abundant ion species were picked for fragmentation, normalized collision energy (NCE) was set to 32 and the isolation window was at 0.7 *m*/*z*. MS2 scans were acquired at 45,000 resolution, fixed first mass 120 *m*/*z*, AGC target value of 3e5 and maximum IT of 86 ms. Dynamic exclusion was set to 30 s and ions with charge state 1, 6 or higher were excluded from fragmentation. For analysis of phosphoproteomic fractions of TMT-labeled samples the liquid chromatography–mass spectrometry parameters were the same, with the exception of MS2 maximum IT that was set to 120 ms.

### TMT raw data search and processing

All TMT mass spectrometry raw files were analyzed together in one MaxQuant (v.2.0.3.0)^66^ run. Data were searched against the human reference proteome (UP000005640) downloaded from UniProt in January 2021 (https://ftp.uniprot.org/pub/databases/uniprot/previous_releases/) and default protein contaminants. TMT correction factors were applied and the minimum reporter precursor intensity fraction was set to 0.5. Fixed modifications were set to carbamidomethylation of C and variable modifications were set to M oxidation and acetylation of protein N-termini. TMT global proteome and phosphopeptides fractions were analyzed in the same MaxQuant run in separate parameter groups using the same settings, except for including also phospho (STY) as a variable modification when searching phosphopeptide fractions. A maximum of five modifications per peptide were allowed. N-terminal acetylation and M-oxidation were used in protein quantification. Only unique and razor peptides were used for protein quantification. Protein FDR was set to 0.01. Protease specificity was set to Trypsin/P. MaxQuant output files were further analyzed in R studio (v.4.1.1). The protein groups file was filtered for reverse hits, potential contaminants and proteins only identified by site. Protein groups were further filtered for at least two peptides and at least one unique or razor peptide. The TMT-based phosphosite table was expanded by multiplicity and reverse database hits and potential contaminants were removed. Corrected reporter ion intensity columns of both tables were log_2_ transformed and normalized by subtraction of the internal standard channel contained in each TMT plex. The resulting TMT ratios were normalized via median-median absolute deviation (MAD) normalization. Before differential expression analysis, data were filtered for detection in more than 49% of cohort samples. For comparing healthy and malignant samples, only MACS-sorted samples were compared. Proteomic results are available in Supplementary Table 6 (global proteome) and Supplementary Table 7 (phosphoproteome).

### Label-free proteomic analysis of cell lines

CD138 MACS sorted and unsorted cell line samples were fractionated online with a 2 h gradient and mass spectrometry data were acquired on a Q Exactive Plus mass spectrometer in data dependent acquisition (DDA) mode (top ten). MS1 scans were acquired at 70,000 resolution, scan range of 350–2000 *m*/*z*, maximum IT of 50 ms and AGCtarget value of 3e6. NCE was set to 26 and the isolation window was at 1.6 *m*/*z*. MS2 scans were acquired at 17,500 resolution, fixed first mass 120 *m*/*z*, AGC target value of 5e4 and maximum IT of 50 ms. Dynamic exclusion was set to 30 s and ions with charge state 1, 6 or higher were excluded from fragmentation. Label-free DDA data were analyzed in MaxQuant 2.0.1.1. using default parameters. The LFQ and match between run options were enabled. Phospho (STY) was included as a variable modification for searching the phosphoproteome data. MaxQuant LFQ intensities were log_2_ transformed and filtered for contaminants, identified by side, as well as valid values (minimum three per experimental group). The missing values were imputed from a normal distribution with a width of 0.3 times the standard deviation in the sample and a downshift of 1.8 from the observed mean. LFQ intensities were median normalized before differential expression analysis and experimental groups (control and MACS) were compared using a two-sided moderated two-sample *t*-test.

UBE2Q1 overexpressing samples were analyzed as described previously using data-independent acquisition (DIA)^67^. Label-free DIA data were searched using DIA-NN 1.8.1 software against the human UniProt reference proteome^68^. The search was performed in library-free mode with the in silico FASTA digest parameter enabled. The peptide length range was set to 7–30, and the precursor charge range was set to 1–4. The *m*/*z* range for precursors was set to 340–1,650, and for fragment ions, it was set to 200–1,800. The rest of parameters were set to default with reannotate and match between run being enabled. LFQ protein intensities from the DIA-NN pg output table were log_2_ transformed and filtered for contaminants and peptides per protein (>1), as well as valid values (>70%). Imputation was performed as described above and resulting intensities were median normalized before differential expression analysis. Experimental groups (empty overexpression vector (empty OE) and UBE2Q1 overexpression (UBE2Q1 OE)) were compared using a two-sided moderated two-sample *t*-test.

### Cell culture

All cell lines were obtained from the American Type Culture Collection (ATCC) or DSMZ German Collection of Microoganisms and Cell Cultures and were maintained in RPMI-1640 medium containing 10% fetal bovine serum (FBS) and supplemented with 1% penicillin/streptomycin and 1% l-glutamine. NCI-H929 cells were cultured in media supplemented with beta-mercaptoethanol and sodium pyruvate, and INA-6 cells were cultured in media supplemented with IL-6. Cells were maintained at 37 °C with 5% CO2 in the humidified atmosphere.

### CRISPR–Cas9 activation screen

Lentiviral plasmid dCAS-VP64_Blast was a gift from Feng Zhang (Addgene plasmid #61425)^69^ and was used to stably transduce MM.1S cells. The human Calabrese CRISPR activation pooled library set A was a gift from David Root and John Doench (Addgene #92379)^70^. Lentivirus was produced using HEK293T cells via transfection of the guide library with pSPAX2 and pMD2.G. Virus titration was performed to achieve a MOI of ~0.3 in MM.1S dCas-VP64 cells. A total of 1 × 10^8^ MM.1S dCas-VP64 cells were transduced, and 3 × 10^7^ cells were collected for baseline comparison. The remaining cells were maintained and the media were refreshed every 3 days. On day 28, all cells were collected for genomic DNA analysis. Genomic DNA extraction was performed with Wizard Genomic DNA Purification Kit (A1120). The guide RNA library was amplified and cleaned up with AMPure XP beads. Library single guide (sg)RNAs were sequenced on a NextSeq 500 instrument (Illumina). The MAGeCK algorithm (https://www.bioconductor.org/packages/release/bioc/html/MAGeCKFlute.html) was utilized for analyzing normalized reads and beta score. The beta score indicates the difference in sgRNA abundance between day 4 and day 28, a high score indicating a survival advantage of the respective gene.

### Generation of UBE2Q1 overexpression cell lines

UBE2Q1 cDNA was cloned into retroviral vector pRSF91-FLAG-GW-IRES-GFP-T2A-Puro via a Gateway reaction. Retroviral vectors containing empty or UBE2Q1 constructs generated in HEK293T cells were used to stably transduce MM cell lines OPM2 and LP-1. Seven days posttransduction, cells were placed under puromycin selection. At the time of analysis, the purity of stable cell lines was 99% GFP fluorescence as determined by flow cytometry.

### Inhibitor treatment and viability assays

NT157 was obtained from SelleckChem (S8228), erdafitinib was purchased from Hölzel Diagnostics (HY-18708). Cells were seeded in 384-well plates with respective treatments and plates were incubated at 37 °C for 96 h. Cell viability readout was measured using CellTiter-Glo Luminescent Cell Viability Assay on a POLARstar Omega plate reader.

### FACS analysis of FCRL2 expression

FCRL2 fluorescence-activated cell sorting (FACS) analysis was performed on primary cells, of 14 samples from patients with MM (13 bone marrow aspirates and one ascitic fluid) and 7 healthy donor samples (6 bone marrow samples and one peripheral blood). All samples contained isolated mononuclear cells and were stained with allophycocyanin (APC) anti-FCRL2 (Miltenyi Biotech, 130-107-439). For myeloma cell identification, we used BV421 anti-BCMA (BioLegend, 357519) and FITC anti-SLAMF7 (BioLegend, 331818). The different subpopulations of immune cells were distinguished by PE anti-CD138 (BD Pharmigen, 552026), FITC anti-CD19, PE anti-CD3 (both from BioLegend, 302206 and 344806) as well as PC7 anti-CD13, PE anti-CD33 and PE anti-CD34 (all from Beckman Coulter, B19714, A07775 and A07776). All antibodies were used in a dilution of 1:40. Data analysis was performed with FlowJo v10. Unstained controls were used to set the gates for the fluorochromes.

### Survival analysis with bootstrapping and risk score calculation with AIC-optimal model

The analysis was restricted to patients with MM treated with lenalidomide in induction and maintenance therapy as well as high-dose melphalan/auto-SCT within DSMM clinical trials (*N* = 100 patients). For each fully quantified protein and phosphopeptide, a continuous variable Cox proportional hazard model for PFS was calculated and resulting *P* values were corrected with Benjamini–Hochberg. We combined the FDR-controlled approach with 1,000-fold bootstrapping to identify the most reproducibly significant proteins in a cohort of the same size randomly sampled with replacement from our data, that is, allowing multiple occurrences of samples in the bootstrap cohort. The 95% confidence interval of *P* values from the bootstrapping was calculated. Proteins with an upper confidence interval of *P* values <0.1 and an FDR <0.1 (*n* = 32) were selected as candidates for the final risk score. A multi-protein Cox PH model was constructed by step-wise addition of optimal proteins based on the Akaike Information Content (AIC), balancing increased model performance versus increased model complexity. The final risk score was calculated on the AIC-optimal multi-protein model, by linear combination of the protein abundance scaled by the model coefficients. This resulted in a protein score containing protein-level information of eight proteins with differing weights. The inclusion of additional proteins or phosphopeptides into the model only led to marginal improvement in the survival prediction accuracy. Differences in survival were analyzed with a log-rank test. For validation, we calculated the protein risk score on untreated myeloma samples analyzed by Kropivsek et al.^21^ based on the provided protein quantifications (‘CD138 cells’ quantification). The term for PDSS2 was omitted from the risk score since it was not quantified in the Kropivsek et al. cohort. No other adaptations of the risk score were employed. Survival curves were stratified by the median risk score of the respective cohort.

### RNA–protein correlation and CNV buffering analysis

For RNA–protein correlation analysis RNAseq samples were filtered for a minimum plasma cell content of 80% and a mapped read count higher than 20 million. Proteome data were collapsed to gene-level information via median and RNA and protein datasets were matched by gene name. Copy number variation (CNV) data were matched with RNA and protein data via the cytogenetic band of the corresponding gene locus. For calculating Pearson correlation across MM samples, the resulting data matrix was filtered for at least ten paired values. To estimate the buffering of CNVs from RNA to protein level we calculated a customized score with the following formula:

$${\mathrm{buffering}}\; {\mathrm{score}}_{g}=\left[\mathrm{corr}\left({\mathrm{RNA}}_{g},{\mathrm{CN}}_{g}\right)-\mathrm{corr}\left({\mathrm{protein}}_{g},{\mathrm{CN}}_{g}\right)\right]\times \left|\bar{{{{\mathrm{CN}}}}_{g}}-2\right|$$

For each gene (*g*) we subtracted the Pearson correlation (corr) of protein to copy number (CN) from the Pearson correlation (corr) of RNA to CN. The resulting delta was corrected with the average copy number effect diverging from a diploid genotype. Pearson correlations and buffering scores were subjected to ssGSEA analysis as described below.

### SsGSEA

The ssGSEA implementation available on https://github.com/broadinstitute/ssGSEA2.0 was used to separately project protein and phosphopeptide abundance changes to signaling pathways. The normalized ratio or fold change matrix was collapsed to gene level information via median and subjected to ssGSEA. For ssGSEA of normalized TMT ratios, the gene set databases containing curated gene sets (C2.all.v7.0.symbols.gmt), oncogenic signature gene sets, (c6.all.v7.0.symbols.gmt) and hallmark gene sets (h.all.v7.0.symbols.gmt) were used. For ssGSEA of RNA to protein correlations, the Kyoto Encyclopedia of Genes and Genomes (KEGG) gene sets (c2.cp.kegg.v7.0.symbols.gmt) were used. For ssGSEA of buffering of CNVs from RNA to protein level, databases containing positional genesets (c1.all.v7.0.symbols.gmt) and KEGG gene sets (c2.cp.kegg.v7.0.symbols.gmt) were used. The following parameters were used for all ssGSEA analyses: sample.norm.type = ‘rank’, weight = 0.75, statistic =‘area.under.RES’, output.score.type = ‘NES’, nperm = 1,000, min.overlap = 10, correl.type = ‘z.score’

### NMF clustering of ssGSEA enrichment scores

Normalized ssGSEA scores of phosphoproteomic data were used as input for NMF with the NMF R package (v.0.23.0)^71^ as previously described^72^. The following parameters were used: *K* = 2:7, method = ‘brunet’, nrun = 50. The cophenetic correlation coefficient was used to evaluate the clustering quality. After determining the optimal factorization rank *k*, we repeated the NMF analysis using 500 iterations with random initializations and performed partitioning of samples into clusters.

### GO term analysis with Metascape

Gene Ontology (GO) term enrichment analysis of a gene list corresponding to proteins regulated in 1q gain not located on chromosome 1q was performed with the Metascape^73^ online tool.

### Integration of Depmap data

Proteins significantly upregulated in myeloma versus healthy samples (<0.1 FDR) or selectively identified in myeloma samples were further filtered for potential therapeutic targets by integrating the depmap CRISPR KO database (gene effect download file^74^). First, genes coding proteins in our candidate list were filtered for median dependency in myeloma cell lines <−0.4 (median dependency of the myeloma therapeutic targets IKZF1 and IKZF3). Common essential genes (DepMap Public 22Q2) were excluded from the target list. In addition, genes were filtered for having a minimum difference of median dependency in myeloma versus median dependency in nonmyeloma cell lines >0.1.

### RNA sequencing data analysis

RNA sequencing data were aligned and quantified with STAR and messenger RNA reads were identified using an in-house analysis pipeline detecting exons in a shuffled order. To increase comparability to TMT data, RNA gene-level transcripts per million (TPM) values were further normalized as described previously^75^. First, TPM gene-level data were normalized via median subtraction (by gene) and, subsequently, each sample was normalized by median-MAD normalization. The normalized data are available in Supplementary Table 2.

### Nanopore DNA sequencing data analysis

After basecalling, the sequenced reads were aligned with minimap2 (ref. ^76^) to the University of California, Santa Cruz (UCSC) hg19 genome reference (https://www.ncbi.nlm.nih.gov/grc) without haplotype specific scaffolds. After conversion of the alignment files (SAMtools v.0.1.19, https://github.com/samtools/) SAM format, (https://samtools.github.io/hts-specs/SAMv1.pdf)) sorting and indexing to binary alignment format (BAM format, https://samtools.github.io/hts-specs/SAMv1.pdf) the copy number profiles were generated with the absolute copy number estimate package^77^ in R (4.2.1, https://cran.r-project.org/) with a bin size of 1 million base pairs. Errors were estimated with ‘maximum absolute error’ and only autosomes were called. The resulting copy number aberrations were reported on to genomic band level to the nearest integer. Ambiguous copy numbers were called by the most prevalent copy number on the particular band. Bands with insufficient reads were marked as NA. For subclonal events, the nearest natural number was chosen, except in the vicinity of two where a deviation threshold of 0.35 was used to maximize the concordance with FISH results.

Ploidy and cellularity (relevant local minimum used) of each sample in absolute copy number estimate were matched to existing FISH data. If FISH data were not available, the profiles were chosen for plausibility, minimizing the number of aberrations and avoiding scaffolds with copy number 0. The processed data are available in Supplementary Table 2. Four additional cases without 9q amplification were assigned to the hyperdiploidy group based on nanopore sequencing

### Validation by single-cell sequencing data

Expression of candidates from the proteomic analysis was further validated with single-cell RNA sequencing data of bone marrow from healthy individuals and patients with MM from Lutz et al.^51^. Uniform manifold approximation and projection (UMAP) plots highlighting normalized expression for genes of interest were generated in R using the FeaturePlot() function from the Seurat package^78^.

### Statistics and reproducibility

No statistical method was used to predetermine sample size, samples were chosen based on availability. As the study focuses on newly diagnosed samples, four TMT labeled samples corresponding to relapse cases were excluded from the analysis. In the TMT plex analyzing healthy cells, carrier channels containing the booster channel and unsorted mononuclear cells were excluded from further analysis; they were present in the TMT plex to increase coverage of low abundant proteins. Patient samples were randomly distributed across TMT plexes. Technical replicates of eight samples were differentially labeled and included in different TMT plexes. Replicates clustered together as expected and had an average Pearson correlation coefficient of 0.8 for global proteome and 0.77 for phosphoproteomic normalized ratios, respectively. We performed four or three biological replicates of cell culture experiments for proteomics or inhibitor treatments, respectively. All attempts of replication were successful and no replicate was excluded from analysis. Differentially expressed proteins were determined with a two-sided moderated two-sample *t-*test (limma package). The resulting *P* values were corrected with the Benjamini–Hochberg method. Drug treatments of each cell line were compared to respective dimethyl sulfoxide (DMSO) controls with a Dunnett’s test. For analyzing CRISPR–Cas9 activation screen data, the MAGeCK maximum-likelihood estimation (MLE) algorithm was applied for the analysis of beta scores and *P* values.

### Reporting summary

Further information on research design is available in the Nature Portfolio Reporting Summary linked to this article.

### Supplementary information

Reporting Summary

Supplementary Table 1 Supplementary Tables: table names and legends contained in the file.

### Source data

Source Data Fig. 1 Statistical source data.

Source Data Fig. 2 Statistical source data.

Source Data Fig. 3 Statistical source data.

Source Data Fig. 4 Statistical source data.

Source Data Fig. 5 Statistical source data.

Source Data Fig. 6 Statistical source data.

Source Data Fig. 7 Statistical source data.

Source Data Extended Data Fig. 1 Statistical source data.

Source Data Extended Data Fig. 2 Statistical source data.

Source Data Extended Data Fig. 3 Statistical source data.

Source Data Extended Data Fig. 4 Statistical source data.

Source Data Extended Data Fig. 5 Statistical source data.

Source Data Extended Data Fig. 6 Statistical source data.

Source Data Extended Data Fig. 7 Statistical source data.

Source Data Extended Data Fig. 8 Statistical source data.

Source Data Extended Data Fig. 9 Statistical source data.

## Data Availability

Data that support the findings of this study have been deposited in the following repositories. Mass spectrometry data have been deposited on PRIDE with the accession numbers PXD038437 and PXD043580. Processed proteomics data of patient samples can be interactively explored at https://myelomaprot.mdc-berlin.de/. RNA sequencing expression data are available at the Gene Expression Omnibus under accession number GSE222727. Previously published microarray data that were reanalyzed here are available under accession code GSE2658 ref. ^34^. Proteomics data were searched against the human reference proteome (UP000005640) downloaded from UniProt in January 2021 (https://ftp.uniprot.org/pub/databases/uniprot/previous_releases/). Source data are provided with this paper. All other data supporting the findings of this study are available from the corresponding author on reasonable request.

## Code availability

The data were processed as described in Methods. All used R packages are public and are freely available online. No new code or mathematical algorithms were generated from this manuscript.
