# Supplementary material for: The proteogenomic landscape of multiple myeloma reveals insights into disease biology and therapeutic opportunities
Source: Nat Cancer. 2024 Jun 28;5(8):1267–84. doi: 10.1038/s43018-024-00784-3 (PMC11358022; doi:10.1038/s43018-024-00784-3)
Supplement: Supplementary file 1 — Reporting Summary [file 43018_2024_784_MOESM1_ESM.pdf]

nature portfolio

Corresponding author(s): Jan Krönke

Last updated by author(s): Dec 2, 2024

Reporting Summary

Nature Portfolio wishes to improve the reproducibility of the work that we publish. This form provides structure for consistency and transparency in reporting. For further information on Nature Portfolio policies, see our Editorial Policies and the Editorial Policy Checklist.

Statistics

For all statistical analyses, confirm that the following items are present in the figure legend, table legend, main text, or Methods section.

n/a	Confirmed	
☐	☒	The exact sample size (*n*) for each experimental group/condition, given as a discrete number and unit of measurement
☐	☒	A statement on whether measurements were taken from distinct samples or whether the same sample was measured repeatedly
☐	☒	The statistical test(s) used AND whether they are one- or two-sided<br>*Only common tests should be described solely by name; describe more complex techniques in the Methods section.*
☐	☒	A description of all covariates tested
☐	☒	A description of any assumptions or corrections, such as tests of normality and adjustment for multiple comparisons
☐	☒	A full description of the statistical parameters including central tendency (e.g. means) or other basic estimates (e.g. regression coefficient) AND variation (e.g. standard deviation) or associated estimates of uncertainty (e.g. confidence intervals)
☐	☒	For null hypothesis testing, the test statistic (e.g. *F*, *t*, *r*) with confidence intervals, effect sizes, degrees of freedom and *P* value noted<br>*Give P values as exact values whenever suitable.*
☒	☐	For Bayesian analysis, information on the choice of priors and Markov chain Monte Carlo settings
☒	☐	For hierarchical and complex designs, identification of the appropriate level for tests and full reporting of outcomes
☐	☒	Estimates of effect sizes (e.g. Cohen's *d*, Pearson's *r*), indicating how they were calculated

Our web collection on statistics for biologists contains articles on many of the points above.

Software and code

Policy information about availability of computer code

Data collection	Proteomics: Q-Exactive HF-X and Exploris 480 (ThermoFisher Scientific)<br>RNA-seq: single end RNA sequencing HiSeq2000 (Illumina)<br>nanopore DNA seq: sequencing was performed on a GridION sequencer (Oxford Nanopore Technologies (ONT) platform)<br>CRISPR/Cas9 activation screen : Library sgRNA was sequenced on a nextSeq 550 System (Illumina)<br>Cell viability: POLARstar® Omega plate reader
Data analysis	Proteomics: raw data was analyzed with MaxQuant (Version 2.0.3.0 for cell culture experiments and 2.0.1.1. for patient samples or with DIANN 1.8.1, processed raw data was further analyzed in R studio (V 4.1.1)<br>The single sample Gene Set Enrichment Analysis (ssGSEA) implementation available on https://github.com/broadinstitute/ssGSEA2.0 was used to separately project protein and phosphopeptide abundance changes to signaling pathways in R. GO term enrichment analysis of a gene list corresponding to proteins regulated in 1q gain not located on chromosome 1q was performed with the Metascape online tool.<br>RNA-seq: alignment with STAR 2.7, quantification with RSEM 1.3.0, analysis with R 4.0.3 GUI 1.73. TPM RNA data was futher normalized with R studio (V 4.1.1) to increase comparability to proteomics data.<br>nanopore DNA seq : sequenced reads were aligned with minimap2. After conversion of the alignment files (samtools v. 0.1.19, https://github.com/samtools/) SAM format, (https://samtools.github.io/hts-specs/SAMv1.pdf)) sorting and indexing to binary alignment format (BAM format, https://samtools.github.io/hts-specs/SAMv1.pdf) the copy number profiles were generated with the absolute copy number estimate (ACE) package in R (4.2.1).<br>CRISPR/Cas9 activation screen: The MAGeCK package (version 0.5.9.4.) was utilized for analyzing normalizing reads and beta-score

For manuscripts utilizing custom algorithms or software that are central to the research but not yet described in published literature, software must be made available to editors and reviewers. We strongly encourage code deposition in a community repository (e.g. GitHub). See the Nature Portfolio guidelines for submitting code & software for further information.

Data

Policy information about availability of data

All manuscripts must include a data availability statement. This statement should provide the following information, where applicable:

- Accession codes, unique identifiers, or web links for publicly available datasets
- A description of any restrictions on data availability
- For clinical datasets or third party data, please ensure that the statement adheres to our policy

Data that support the findings of this study have been deposited in the following repositories. Mass spectrometry data have been deposited on PRIDE with the accession number PXD038437 and PXD043580. Processed proteomics data of patient samples can be interactively explored at https://myelomaprot.mdc-berlin.de/ . RNAseq expression data is available at GEO under the accession number GSE222727. Source data is provided with the article. Previously published microarray data that were re-analysed here are available under accession code GSE265834. Proteomics data was searched against the human reference proteome (UP000005640) downloaded from UniProt in 01/2021 (https://ftp.uniprot.org/pub/databases/uniprot/previous_releases/). Source data for all Main Figures and Extended Data Figures have been provided as Source Data files. All other data supporting the findings of this study are available from the corresponding author on reasonable request.

Research involving human participants, their data, or biological material

Policy information about studies with human participants or human data. See also policy information about sex, gender (identity/presentation), and sexual orientation and race, ethnicity and racism.

Reporting on sex and gender	only biological sex is reported. Information on gender was not collected
Reporting on race, ethnicity, or other socially relevant groupings	Information on race, ethnicity or other socially groupings was not collected.
Population characteristics	Bone marrow samples from 138 patients encompassing treatment-naive newly diagnosed multiple myeloma (N=114), plasma cell leukemia (N=17), and MGUS (N=7). 100 of the newly diagnosed patients were treated within one of three consecutive trials of the DSMM XII, XIII, XIV (NCT00925821, NCT01090089, NCT01685814) and were scheduled to receive a lenalidomide-based induction therapy, high-dosage melphalan with autologous stem cell transplantation and lenalidomide maintenance therapy and had available outcome data . Patient characteristics are summarized in Table S1.
Recruitment	Patients were included based on the availability of a bone marrow/ blood sample with more than 75% tumor cell content and/ or CD138-MACS sorting, genetics performed within standard routine (FISH) and clinical data.<br>Of the 138, 89 were CD138+ enriched using MACS immediately post-PBMC isolation with beads linked to a CD138-specific antibody (#130-051-301, Miltenyi). The remaining 49, not enriched via MACS, were chosen for their >75% plasma cell content, showing an average CD138+ purity of 85%. Despite tumor purity potentially influencing proteomic composition, our major findings are consistent across sorted and non-sorted samples.
Ethics oversight	All patients provided written informed consent according to the Declaration of Helsinki and the study was approved by the responsible ethic committees Ulm University (136/20, 307/08) and Charite Universitätsmedizin Berlin (EA2/142/20). The DSMM trial was approved by the ethics committee of Würzburg Universit (DSMM XII: 2008-000007-28, DSMM XIV: 145-11). All donors pf healthy bone marrow material provided written informed consent according to the Declaration of Helsinki and the study was approved by the responsible ethic committee Charite - Universitätsmedizin Berlin (EA4/115/21).

Note that full information on the approval of the study protocol must also be provided in the manuscript.

Field-specific reporting

Please select the one below that is the best fit for your research. If you are not sure, read the appropriate sections before making your selection.

☒ Life sciences ☐ Behavioural & social sciences ☐ Ecological, evolutionary & environmental sciences

For a reference copy of the document with all sections, see nature.com/documents/nr-reporting-summary-flat.pdf

Life sciences study design

All studies must disclose on these points even when the disclosure is negative.

Sample size	Available bone marrow biopsied from multiple myeloma MGUS and plasmacell leukemia patients were analyzed. No statistical method was used to predetermine the sample size that was limited by the number of samples provided with sufficient protein material.
Data exclusions	Proteomics: Technical replicates to assess reproducibility of TMT measurments were not further included in downstream analysis. Carrier channels in TMT channel setup (see table S11) that were used to boost identification of proteins were not further analyzed. for comparison of healthy and disease bone marrow, only MACS sorted samples were analyzed to increase comparability<br>RNAseq: For RNA-protein correlation analysis, a stringent data quality filter was first applied and RNAseq samples were filtered for a minimum plasmacell content of 80% and a mapped read count higher than 20 million. These cutoffs were chosen in a way that further stringency did

not improve overall RNA-protein correlation. As RNA is less stable than protein, we expect a higher degree of variability in RNAseq data quality. All sequenced RNA samples passed the initial RNA quality filter specified in the TruSeq Stranded Exome RNA Kit (DV200 > 30%)

Replication	Technical replicates of 8 patient samples were included in different TMT plexes to assess the reproducibility of TMT based proteomics. All attempts of replication were successful; replicates clustered together as expected and had an average Pearson correlation coefficient of 0.8 for global proteome and 0.77 for phosphoproteomic normalized ratios, respectively. For cell culture experiments, we performed four (proteomic experiments in cell lines) or three (inhibitor experiments) independent biological replicates and all attempts at replication were successful.
Randomization	Randomization for clinical data was not applicable because this study was performed retrospectively where samples were chosen based on availability. TMT channels for proteomics data measurment were randomly assigned
Blinding	We performed proteogenomic analysis of available retrospective samples. No blinding was applied

Reporting for specific materials, systems and methods

We require information from authors about some types of materials, experimental systems and methods used in many studies. Here, indicate whether each material, system or method listed is relevant to your study. If you are not sure if a list item applies to your research, read the appropriate section before selecting a response.

Materials & experimental systems

n/a	Involved in the study
☐	☒ Antibodies
☐	☒ Eukaryotic cell lines
☒	☐ Palaeontology and archaeology
☒	☐ Animals and other organisms
☐	☒ Clinical data
☒	☐ Dual use research of concern
☒	☐ Plants

Methods

n/a	Involved in the study
☒	☐ ChIP-seq
☐	☒ Flow cytometry
☒	☐ MRI-based neuroimaging

Antibodies

Antibodies used	MACS sorting: CD138-specific (#130-051-301), CD19-specific and CD34-specific microbeads, human (all Miltenyi, Cologne, Germany), FACS analysis: FCRL2:Miltenyi Biotech APC CD307b (FcRL2) Antibody, anti-human, REAfinity™, Clone: REA474, Catalogue #: 130-107-439; CD138: BD Pharmingen™ PE Mouse Anti-Human CD138, Clone: MI15, Catalogue #: 552026; BCMA: BioLegend® Brilliant Violet 421™ anti-human CD269 (BCMA) Antibody, Clone: 19F2, Catalogue #: 357519; SLAMF7: BioLegend® FITC anti-human CD319 (CRACC) Antibody, Clone: 162.1, Catalogue #: 331818; CD19: BioLegend® FITC anti-human CD19 Antibody, Clone: HIB19, Catalogue #: 302206; CD3: BioLegend® PE anti-human CD3 Antibody, Clone: SK7, Catalogue #: 344806;<br>CD33: Beckman Coulter PE CD33, Clone: D3HL60.251, Catalogue #: A07775;<br>CD34: Beckman Coulter PE CD34, Clone: 581, Catalogue #: A07776; CD13: Beckman Coulter PC7 CD13, Clone: 366, Catalogue #: B19714
Validation	All antibodies used in this study were procured directly from manufacturers and were validated by the manufacturers for both antigen specificity and reactivity with human cells using flow cytometry. Briefly, BioLegend® confirmed specificity through single- or multi-color flow cytometry analysis of human peripheral blood lymphocytes (anti-CD3, anti-CD19, anti-SLAMF7) or the human myeloma cell line U266 (anti-BCMA); Miltenyi Biotech validated their anti-FCRL2 antibody on human peripheral blood mononuclear cells and conducted an epitope competition assay additionally; Beckman Coulter conducted validation on human whole blood samples, comparing results with a reference reagent; and BD Pharmingen™ validated their anti-CD138 antibody on the U266 cell line. Comprehensive details on the extended validation procedures for each antibody are available in the technical datasheets and references provided by the respective manufacturers. All antibodies utilized in this study exhibited expected staining results in accordance with the existing literature.

Eukaryotic cell lines

Policy information about cell lines and Sex and Gender in Research

Cell line source(s)	Multiple myeloma cell lines MM.1S, LP-1, OPM2, NCI-H929, AMO-1, INA-6, and JJN3, as well as HEK293T cells were purchased from DSMZ or ATCC.
Authentication	Cell line authentification was performed via short tandem repeat(STR) profiling. Molecular markers were compared against STR profile database DSMZ CellDive to confirm correctness of cell lines. Regular authentification was performed on all cell lines.
Mycoplasma contamination	All cell lines were tested negative for Mycoplasma contamination.
Commonly misidentified lines (See ICLAC register)	No commonly misidentified cell lines were used.

Clinical data

Policy information about clinical studies

All manuscripts should comply with the ICMJE guidelines for publication of clinical research and a completed CONSORT checklist must be included with all submissions.

Clinical trial registration	NA
Study protocol	NA
Data collection	NA
Outcomes	NA

Flow Cytometry

Plots

Confirm that:

- [x] The axis labels state the marker and fluorochrome used (e.g. CD4-FITC).
- [x] The axis scales are clearly visible. Include numbers along axes only for bottom left plot of group (a 'group' is an analysis of identical markers).
- [ ] All plots are contour plots with outliers or pseudocolor plots.
- [x] A numerical value for number of cells or percentage (with statistics) is provided.

Methodology

Sample preparation	All samples contained isolated mononuclear cells and were stained with APC anti-FCRL2 (Miltenyi Biotech, 130-107-439). For myeloma cell identification, we used BV421 anti-BCMA (BioLegend®, 357519) and FITC anti-SLAMF7 (BioLegend®, 331818). The different subpopulations of immune cells were distinguished by PE anti-CD138 (BD Pharmigen™, 552026), FITC anti-CD19, PE anti-CD3 (both from BioLegend®, 302206 and 344806) as well as PC7 anti-CD13, PE anti-CD33 and PE anti-CD34 (all from Beckman Coulter, B19714, A07775 and A07776). All antibodies were used in a dilution of 1:40.
Instrument	Beckman Coulter CytoFLEX S
Software	Flow Jo_v10.6.2, Beckman Coulter CytExpert v2.4
Cell population abundance	Cell sorting was not performed.
Gating strategy	Cells were gated based on FSC-H/SSC-H. Single cells were gated with FSC-H/FSC-A followed by SSC-H/SSC-A. Multiple myeloma cells were defined as BCMA+ and/or SLAMF7+. Boundaries for presence of the fluorochromes were defined by gating against unstained controls.

- [x] Tick this box to confirm that a figure exemplifying the gating strategy is provided in the Supplementary Information.
